# Reciprocal recombination genomic signatures in the symbiotic arbuscular mycorrhizal fungi *Rhizophagus irregularis*

**DOI:** 10.1371/journal.pone.0270481

**Published:** 2022-07-01

**Authors:** Ivan D. Mateus, Ben Auxier, Mam M. S. Ndiaye, Joaquim Cruz, Soon-Jae Lee, Ian R. Sanders

**Affiliations:** 1 Department of Ecology and Evolution, University of Lausanne, Lausanne, Switzerland; 2 Laboratory of Genetics, Wageningen University, Wageningen, The Netherlands; Institute for Sustainable Plant Protection, C.N.R., ITALY

## Abstract

Arbuscular mycorrhizal fungi (AMF) are part of the most widespread fungal-plant symbiosis. They colonize at least 80% of plant species, promote plant growth and plant diversity. These fungi are multinucleated and contain either one or two haploid nuclear genotypes (monokaryon and dikaryon) identified by the alleles at a putative mating-type locus. This taxon has been considered as an ancient asexual scandal because of the lack of observable sexual structures. Despite identification of a putative mating-type locus and functional activation of genes related to mating when two isolates co-exist, it remains unknown if the AMF life cycle involves a sexual or parasexual stage. We used publicly available genome sequences to test if *Rhizophagus irregularis* dikaryon genomes display signatures of sexual reproduction in the form of reciprocal recombination patterns, or if they display exclusively signatures of parasexual reproduction involving gene conversion. We used short-read and long-read sequence data to identify nucleus-specific alleles within dikaryons and then compared them to orthologous gene sequences from related monokaryon isolates displaying the same putative MAT-types as the dikaryon. We observed that the two nucleus-specific alleles of the dikaryon A5 are more related to the homolog sequences of monokaryon isolates displaying the same putative MAT-type than between each other. We also observed that these nucleus-specific alleles displayed reciprocal recombination signatures. These results confirm that dikaryon and monokaryon isolates displaying the same putative MAT-type are related in their life-cycle. These results suggest that a genetic exchange mechanism, involving reciprocal recombination in dikaryon genomes, allows AMF to generate genetic diversity.

## Introduction

Arbuscular mycorrhizal fungi are plant endosymbionts, forming symbioses with most plant species, promoting plant growth [1], plant community diversity [2, 3] and affect how plants cope with biotic [4] or abiotic stresses [5]. As a consequence, they are widely used in agriculture [6]. In a single agricultural field, the presence of at least 17 different genotypes of *Rhizophagus irregularis* displaying spatial genetic structure have been detected [7, 8]. Furthermore, these *R*. *irregularis* isolates display different levels of within isolate genetic diversity [9], which has been reported to produce differential effects on plant growth [10]. Understanding how genetic variability is generated in AMF, is important because it could be harnessed to generate genetic variants that could be beneficial for their plant hosts [11].

AMF are part of the Glomeromycotina subphylum [12], which fossil records date to at least ~400 Million years ago [13]. They are coenocytic (without septa separating otherwise adjacent compartments), their hyphae harbor hundreds of nuclei within the same cytoplasm [14] and no single-nucleus state has been recorded. The nuclei of these fungi have been reported as haploid [15–18]. This group of fungi has been previously considered as an ancient asexual scandal [19], due to low morphological diversification and the absence of observable sexual structures. However, different reports suggest that sexual reproduction could be possible in AMF since these fungi contain a complete meiosis machinery [20]. A putative mating-type determining locus (MAT) has been proposed [16], population genetic data suggests the existence of recombination in AMF populations [21] and activation of genes related to mating has been detected when different isolates of the same species co-exist in plant roots [22, 23].

Several *R*. *irregularis* isolates issued from the same geographic location have been reported to have one haploid nuclear genotype (monokaryon: isolates A1,B12 and C2) and two haploid nuclear genotypes (dikaryon: isolates A4, A5, C3 and G1) [16, 24–26], evidencing that monokaryon and dikaryon isolates co-exist in the same location. Single nucleotide polymorphisms (SNP) profiles from single-nucleus from dikaryon isolates (A4, A5 and SL1) cluster into two genetically different groups, that are associated with the identity of a putative MAT-locus [24]. This demonstrates that the presence of two copies of the putative MAT-locus is a reliable marker of the dikaryon state.

Like most fungi, AMF can undergo anastomosis, the fusion of hyphae. Through these connections, bi-directional flow of cytoplasm has been observed between genetically different AMF individuals [27]. Via anastomosis, the transfer of genetic material between vegetative compatible isolates (parasexuality) has been suggested as a mechanism of maintenance of genetic diversity in the absence of sexual recombination [28]. In fungi, hyphal fusion between different individuals leads to cell death, however, non-self vegetative compatibility has also been observed in AMF when different isolates form perfect hyphal fusions [29]. In the model species *Aspergillus nidulans* parasexuality involves fusion of two haploid nuclei, mitotic recombination and haploidization of the diploid nuclei by i.e. chromosome loss [30]. In consequence, a genomic signature of parasexuality is gene-conversion where there is a loss of heterozygosity. Although sexual reproduction can also produce signatures of gene-conversion, sexual reproduction also involves meiotic recombination and allele segregation on reciprocal products [31].

The existence and relevance of sexuality and/or parasexuality for the evolution of AMF remains unknown [32]. It has been hypothesized that monokaryon isolates could fuse to form dikaryon isolates during the AMF life-cycle [16]. However, it is still unclear whether the transition between a dikaryon and monokaryon life stages involves a sexual event involving meiotic recombination, or a parasexual event.

In the absence of stable transformation methods in AMF [33], analyses of continuous genomic sequences (haplotype analyses) could be an important resource to identify genomic signatures of recombination in dikaryon isolates. Nucleus-specific alleles from dikaryon isolates (identified through haplotype phasing) can then be compared to orthologous sequences of related monokaryon isolates (that share the same MAT-type) and could allow to identify genomic signatures of a sexual event, involving meiotic recombination, or a parasexual event which results in the loss of heterozygosity or gene conversion.

AMF genomes display large gene duplication events [18], which make it difficult to distinguish nucleus-specific sequences (orthologs) from sequence duplications (paralogs) in dikaryon isolates. The analysis of nucleus-specific sequences could be used to better differentiate orthologs from paralogs. The identification of nucleotype specific sequences in dikaryon isolates could be made by: 1) Drop in coverage analyses: use of short-read sequences to identify genome-wide copy number variation. These analyses consist in obtaining the read depth, or coverage, after mapping the reads to a genome assembly and identify changes in coverage across the genome [34]. A drop in coverage could represent that the sequences are nucleotype specific. In AMF, a drop in coverage analysis was used to originally identify a putative MAT-locus [16]. 2) Analyzing sequences issued from long-reads sequencing platforms to identify haplotypes and, consequently, nucleotype-specific sequences in fungal dikaryon isolates [35].

Here, we demonstrated that AMF dikaryons display reciprocal recombination genomic signatures by analyzing nucleotype-specific sequences in dikaryon (A5) and monokaryon isolates (A1-C2). In this study we used publicly available data of bulk whole genome short-reads sequencing, single-nucleus short-read sequence data and long-read bulk sequence data to identify nucleotype-specific sequences in dikaryon isolates. We identified regions displaying drops in coverage in short-read whole genome sequence data. In these regions we detected the presence of genes that have two alleles in the dikaryon isolates and one allele in the respective monokaryons. We then confirmed independently, with short-read genome sequence data from single-nucleus, that in dikaryon isolates, different nuclei have different alleles and that they are not always associated to the nucleus genotype (putative MAT identity), evidencing a reciprocal recombination genomic signature. Finally, we validated the analysis, by evaluating long-read genome sequence assemblies and confirmed that A5 dikaryon display genomic signatures of reciprocal recombination.

## Materials and methods

### Source data

We used public-available sequence reads, genome assemblies and annotations of isolates A1, A4, A5, C2 of *R*. *irregularis* for this study, including data from bulk-isolate and single-nucleus sequencing (S1 Table). We used short-read whole genome assemblies from isolates A1, A4, A5 and C2 [16]. We used single-nucleus raw short-reads to generate genome assemblies from individual nuclei of isolates A1, A5 and C2 [24]. We also analyzed long-read genome assemblies of isolates A1, A5 and C2 [36].The long-read genome assembly of the dikaryon isolate A5 is a phased assembly, where the contigs are divided in two parts, the primary assembly and the haplotig assembly. We downloaded the sequence reads from the sequence read archive (SRA) using the SRAtoolkit software with the prefetch and fastq-dump tools [37].

### Coverage analysis on bulk sequencing short-reads

We first trimmed the bulk sequence reads using Trim Galore! [38] with the default parameters. We then used BWA [39] to index the reference genome assemblies and BWA mem -M [39] to map the reads to the reference whole-genome assemblies. We mapped the reads coming from a given isolate to the reference genome assembly of the same isolate (i.e. reads A1 mapped to reference A1). We then kept the reads that display a mapping quality of at least 30. We used the genomecov tool from bedtools [40] to calculate the coverage for each position. We then created a ready-to-use algorithm that detects genome-wide drop in coverage analysis in whole-genome data (S1 File). The algorithm divides the data in portions of 50kb. Then, with a sliding window approach consisting of windows of 400bp and steps of 100bp, the algorithm searches for drops in coverage of 0.3–0.6 times lower than the median coverage of the entire genome and that with a minimum length of 1000bp (Please refer to S1 File for the algorithm specifications implemented in the R programming language). We then further filtered the regions exhibiting a drop in coverage by keeping only the regions that display an average of 1.25 coverage difference between the neighboring regions and the drop in coverage region.

### Gene detection in coverage drops

We identified all the genes located within genomic regions that presented a drop of sequencing coverage. We used the ‘intersect’ command from the BEDTools suite with the existing gene annotations corresponding to each *R*. *irregularis* isolate (GTF format) and their query regions with drops in coverage (BED format) to identify overlapping genes [40]. Genes in scaffolds smaller that 1kb were not considered for further analyses.

### *de novo* single-nucleus assemblies

We trimmed the single-nucleus raw reads by using TrimGalore-0.6.0 [38] with default parameters. After trimming, we performed single-nucleus *de novo* assemblies with SPAdes v3.14 [41] with the following parameters: -k 21,33,55,77—sc—careful—cov-cutoff auto. The resulted single-nucleus genome assemblies were used for further analysis. The length, number of contigs and N50 value of the *de novo* assemblies was evaluated with quast- 5.1.0rc1 with default parameters [42].

### Identification of genes in genome assemblies

To identify the position of gene sequences on the different genome assemblies, we first extracted a query sequence. We then used the console NCBI+ blast suite [43] to blast the query against the desired target. In the case of the putative MAT-locus, we used the homeodomain genes HD2 and HD1-like as query (HD2:KT946661.1, HD1-like: KU597387 from isolate A1). For further downstream analyses, we extracted the sequences from the genome assemblies by using the blastdbcmd command from the NCBI+ suite. We used a reciprocal blast approach to identify the gene sequences corresponding between the whole genome sequence data and the single-nucleus data. We considered the best hits by evaluating the % identity, mismatches, e-value and bitscore. We used the reciprocal blast in the comparison between gene sequences in the short-reads bulk assemblies and the single nucleus assemblies. We do not expect that a high gene copy number plays a role at this step because we worked with highly filtered data. i.e. we are sure that the genes to compare display two copies in the dikaryon and 1 in the monokaryon. So, from the resulting blast output we keep only the best match of the sequence.

### Orthology inference

We used Orthofinder 2.3.11 [44] to identify orthologs of genes found inside the drop in coverage regions within the same isolate. We also identified orthologs in the long-read assemblies and identified single copy orthologs in isolates A1, C2, the primary assembly of A5 and the haplotig assembly of A5. We used the orthogroups output from Orthofinder for the different analyses.

### Synteny plots

We compared genomic regions by performing synteny plots computed with EasyFig2.2.3 [45]. We provide full Genbank files of each contig to compare genomic regions to each other. The software executes a blast comparison between the regions to determine their homology. We used the default parameters for the blast. The figure result can be modified by using the tools on the image menu.

### Genetic distance between nucleus-specific haplotypes on short-reads data

Coding sequences for the 12 confirmed nucleotype-specific genes were extracted using the Blast+ command line blastdbcmd tool [43]. The sequences were then aligned with MAFFT [46] using the—auto option. Then, the ape package [47] of R was used to calculate the pairwise distance between the 4 alleles (2 from A5, and 1 each from A1 and C2).

### Recombination detection in short-reads data

We compared the sequences from drop in coverage regions from both nuclei specific haplotypes of isolate A5 and isolates A1 and C2 to detect if isolate A5 display recombination events between the two putative parental isolates. After identification of the syntenic region among the different isolates, we aligned the sequences with MAFFT [46] and evaluated whether the sequence of one of the nucleotype of isolate A5 was similar to A1 and the other similar to C2.

### Recombination detection in long-reads data

We identified genomic regions in isolate A5 that display long phased sequences containing more than 10 genes. We then identified single-copy orthologs in the monokaryon isolates A1, C2 and on the phased assembly of isolate A5 (primary and haplotig assemblies). We then visualized the phylogenetic relation of each single-copy ortholog among the four genome assemblies by using the gene tree output Orthofinder [44]. We considered a sequence as recombined when within the same continuous phased sequence, we observed that an allele of the A5 primary assembly clusters with an allele of isolate A1, and then a contiguous gene of the same A5 primary assembly clusters with the allele of isolate C2. We did not compare phased sequences separated by unphased sequences because this could lead to artificial recombination signals.

### Phylogenetic analyses

We used MEGA-X [48] for the different phylogenetic reconstructions shown in the study. We find the best DNA models describing the relation between the sequences. Finally, we used a maximum likelihood phylogeny reconstruction with 100 bootstraps to infer the phylogenetic relation among the samples. In several cases, we were not able to perform maximum likelihood phylogenies because of the low number of samples to compare, so UPGMA trees were done instead. Phylogenetic reconstructions of the different orthologous groups on Fig 4 where produced by the Orthofinder software.

## Results

### Drop in coverage analysis on bulk short-reads genome assemblies reveals potential nucleotype-specific haplotypes

Previously, a drop in coverage analysis of selected regions was used for the identification of a putative MAT-locus in *R*. *irregularis* [16]. To perform a similar analysis across the entire genome, we developed a script (S1 File) that allows us to identify genome-wide drop in coverage events (for the accessions of raw data and genome assemblies used in this study see S1 Table).

**Fig 1:**
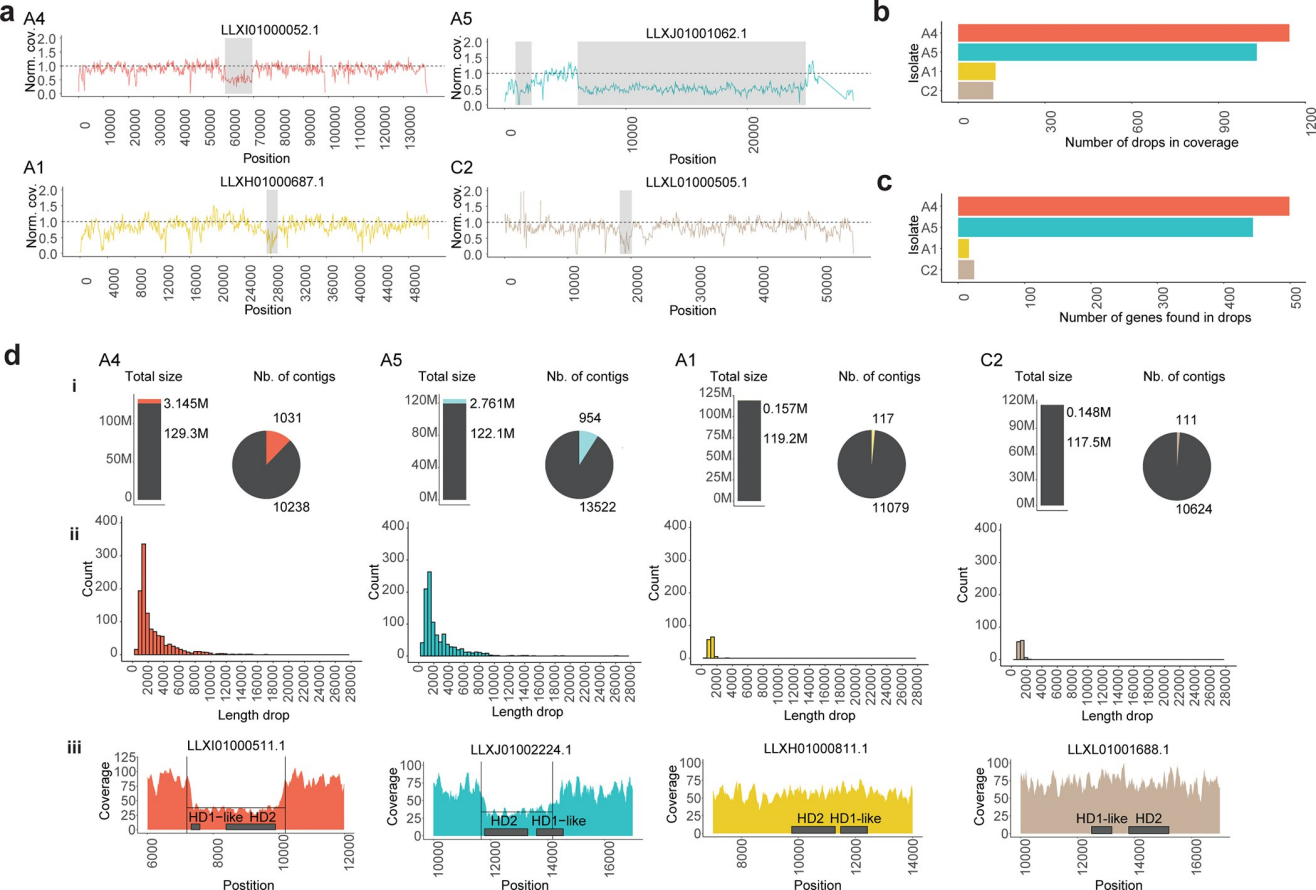
Drop in coverage events in isolates A4, A5, A1 and C2. **a,** Examples of drop in coverage events. We plotted the normalized coverage (y) per position (x). Grey rectangles represent the region detected by the algorithm. The horizontal dashed line represents the normalized coverage. **b,** Number of regions showing a drop in coverage that were detected in each isolate. **c,** Number of genes found in the regions showing a drop in coverage in each isolate. **d,** Summary statistics of regions showing a drop in coverage: i, proportion of total length of regions showing a drop in coverage and proportion of contigs that contain regions with a drop in coverage. ii, Histogram representing the lengths of identified regions where a drop in coverage was detected. iii, Coverage plot on the putative MAT-locus. Drop in coverage was detected in isolates A4 and A5 but not in A1 and C2.

We identified drops in sequencing coverage in 4 different isolates of *R*. *irregularis* which are reported to be dikaryons (A4 and A5) and monokaryons (A1 and C2) (Fig 1, S2 Table). The number of coverage drops and genes inside the drop in coverage regions were different between dikaryons (A4-A5) and monokaryons (A1-C2) (S3 Table). These results indicated that dikaryotic isolates displayed more heterozygous regions than the monokaryons, suggesting that the genes present within the regions showing a drop in coverage are potential candidates for highly divergent, nucleotype-specific alleles in dikaryons. Confirming the reliability of our approach, we detected the expected drop in coverage in the putative MAT-locus region in isolates A4 and A5 but not in isolates A1 and C2 (Fig 1D).

**Fig 2:**
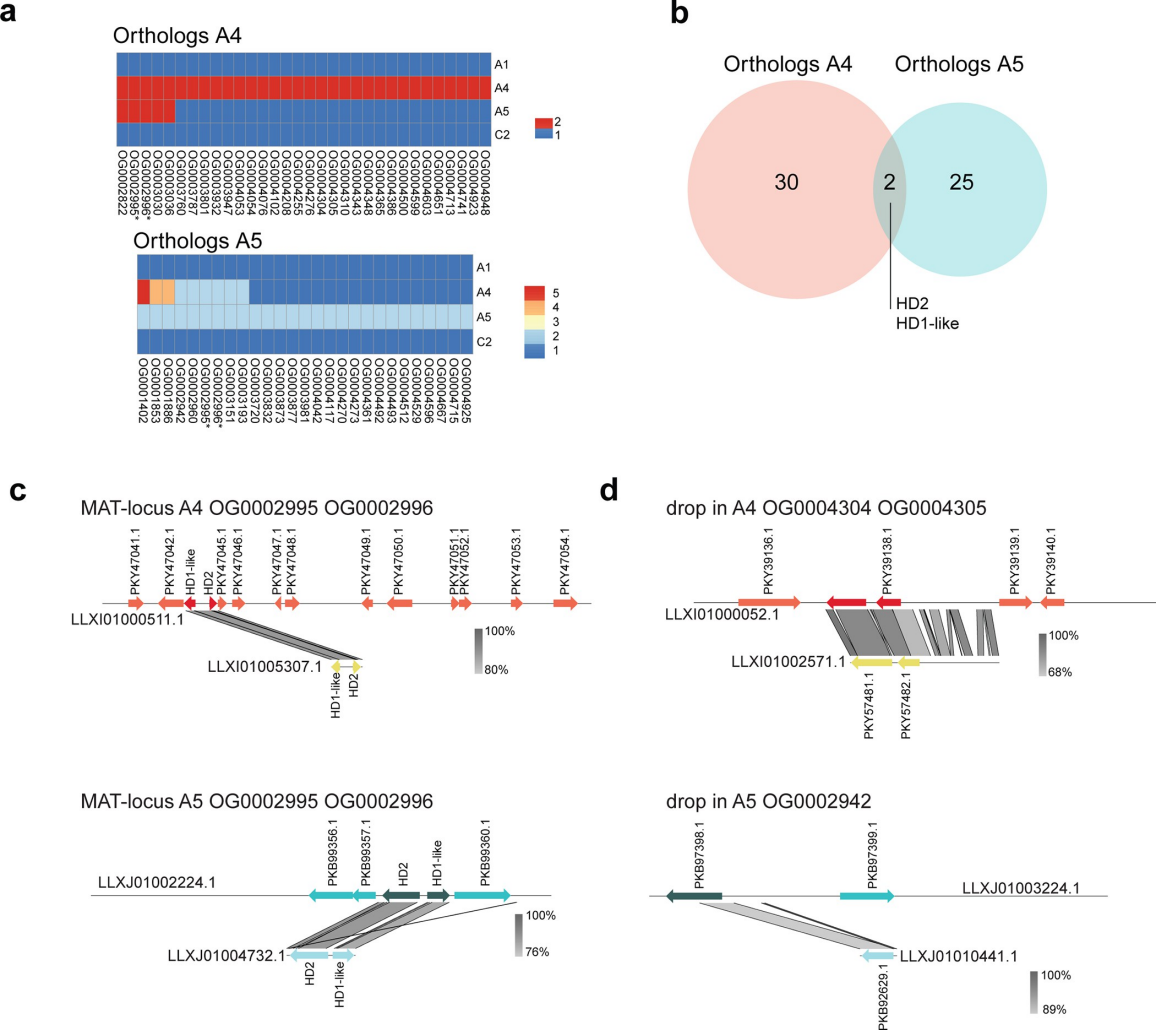
Identification of orthologs of genes present in regions showing a drop in coverage. **a,** Orthologous groups that display two or more genes in dikaryons and only a single gene in monokaryons. This analysis was performed independently on isolate A4 and isolate A5. * orthologous groups where HD2 and HD1-like genes are present. **b,** Venn diagram representing the number of shared orthologous groups within drop in coverage regions between isolates A4 and A5. Only two orthologous groups were shared between the isolates, they contain the putative MAT-locus genes HD2 and HD1-like. **c,** Synteny plot between the two contigs containing the different alleles of the putative MAT-locus of isolates A4 and A5. **d,** Synteny plot between the two contigs containing different alleles of other orthologous genes. Please note that the synteny figures are made from the public available annotations of each genome assembly. Differences in size of open-reading frames (ORF) among isolates are due to differences in detection of ORF on each isolate and likely could be the result of the annotation process.

One cause for a drop in coverage could be copy number variation between the nucleotypes in a dikaryon. To test for this, we inferred orthologous gene families among the different isolates to identify if the genes present in the drop in coverage regions displayed more than one copy in their own genome. We used the gene annotation available for each isolate and inferred the orthology of all the genes present in each genome. Many orthologous groups had more than one copy within each isolate (A4: 20%, A5: 18%, A1: 17% and C2: 19%; Fig 1 and S1 Fig, S4 Table) consistent with the high reported incidence of paralogs in these fungi [18]. We further identified orthologous gene families of genes detected in drop in coverage in isolates A4 and A5 independently. Under the assumption of a monokaryon-dikaryon genome organization in *R*. *irregularis*, to avoid the confounding effect of duplications and reduce the complexity of the dataset, we retained only the orthologous groups that are present in the drop in coverage regions and that display two copies in the dikaryon isolates (A4, A5) and a single copy in the monokaryons (A1, C2) (Fig 2A, S5 Table). In the regions where a drop in coverage was detected, we identified 32 orthologous groups that are present with two copies in isolate A4 and only a single copy in isolates A1 and C2. We also identified 27 orthologous groups in isolate A5 that display two copies. Only two orthologous groups were common between the two isolates: namely, HD2 and HD1-like which are part of the putative MAT-locus in *R*. *irregularis* (Fig 2B). As reported in Ropars *et al*., we observed that the two copies of the putative MAT-locus in the dikaryons were located in different contigs. One copy of HD2 and HD1-like genes were present in a long contig of the genome assembly, while the second copy was present in a much shorter contig (Fig 2C). We observed the same pattern for the other orthologous groups, where the second copy was always present in a second shorter contig (for several examples see Fig 2D).

To further sort paralogous genes from true orthologs, we performed a synteny analysis to compare the genomic location of the presumed orthologous genes among isolates. We identified that 12 out of 32 predicted orthologs in A4 and 16 out of 27 predicted orthologs in A5, were located in the same genomic location on the different isolates, suggesting that they should be considered as orthologs (S5 Table, for examples of inferred orthologs and paralogs see Fig 2, S2 Fig).

Hence, in the whole genome assemblies of dikaryon isolates, two divergent alleles were assembled into different scaffolds; one longer scaffold containing neighboring regions and a shorter scaffold without the neighboring regions. Given that the nuclei are haploid in the dikaryon isolates, two possibilities are consistent with this previous fact: The two copies could be present within the same or in different nuclei.

### Drop in coverage signatures represent nucleotype-specific sequences

**Fig 3:**
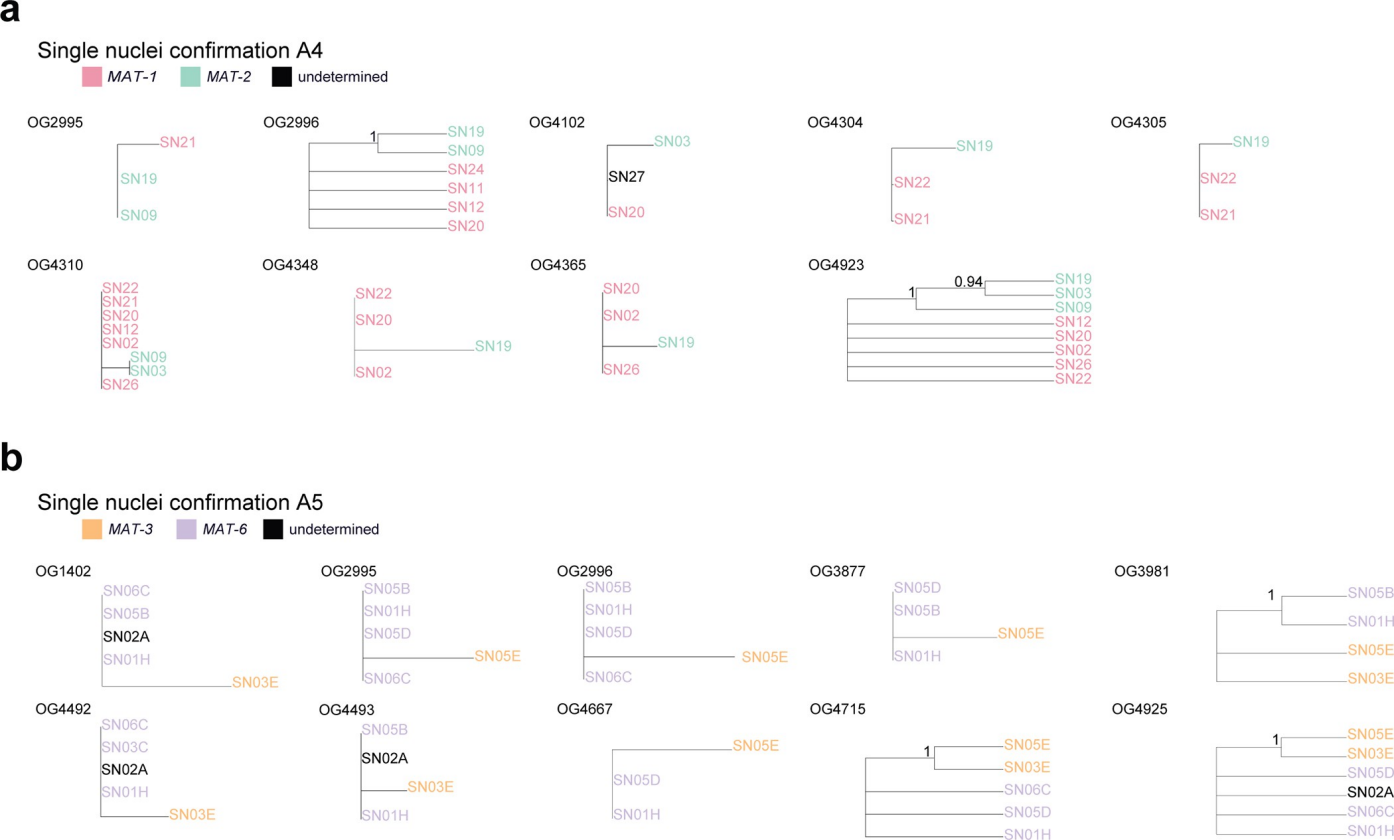
Single-nucleus sequence data confirms that genes contained in regions where a drop in coverage was observed are nucleotype-specific. Phylogenetic reconstruction of single-nucleus for genes found in regions where a drop in coverage was detected in A4 and A5 isolates. The genes are named by their membership to the orthologous groups previously defined. Branch support consisting of 100 bootstraps is shown. When only sequences from three nuclei were included, we performed an UPGMA hierarchical clustering. **a,** data for nuclei from A4 isolate. **b,** data for nuclei from A5 isolate.

To confirm that genes found inside drop in coverage regions are nucleotype-specific, we used sequencing reads of individual nuclei of dikaryon isolates A4 and A5 [24] to produce *de novo* single-nucleus assemblies. The *de novo* assemblies were very fragmented and incomplete (Fig 3, S3A and S3D Fig, S6 Table) and their utilization was highly limited. This limitation resulted in the inability to identify some genes and some complete gene sequences. However, a reciprocal blast approach between the whole genome assembly and the single-nucleus assemblies allowed us to detect sequences in the single-nucleus assemblies corresponding to the genes detected in the whole genome assemblies.

We tested in the dikaryons if the genes identified in the drop in coverage regions were present in the form of different alleles in different single nuclei by using a reciprocal blast approach. We confirmed 9 orthologous genes to be nucleotype-specific in isolate A4 and 12 orthologous genes in isolate A5 (S7 Table). We found that the population of nuclei clustered in two groups that corresponded to the identity of the putative MAT-locus contained in each nucleus (Fig 3). This result confirms that nucleotype-specific alleles in dikaryon isolates can be identified based on genes found in drop in coverage regions and that are represented by a duplication within the genome assembly.

### Nucleotype-specific alleles from A5 share a more recent evolutionary origin with monokaryon isolates A1 and C2 than among them

The origin of dikaryon isolates could be investigated through comparisons of monokaryon isolates that display the same alleles of the putative MAT-locus, to those found in the dikaryons (Isolates A5:MAT-3/MAT-6; A1:MAT-3; C2: MAT-6). A phylogenetic reconstruction of the putative MAT-locus suggests that MAT-3 from isolates A1 and A5 are more closely related than MAT-6 from isolates C2 and A5 [16]. Furthermore, a phylogenetic reconstruction of several *R*. *irregularis* isolates, based on thousands of SNPs, indicated that isolate A5 is more closely related to isolate A1 than to isolate C2 [9, 49].

**Fig 4:**
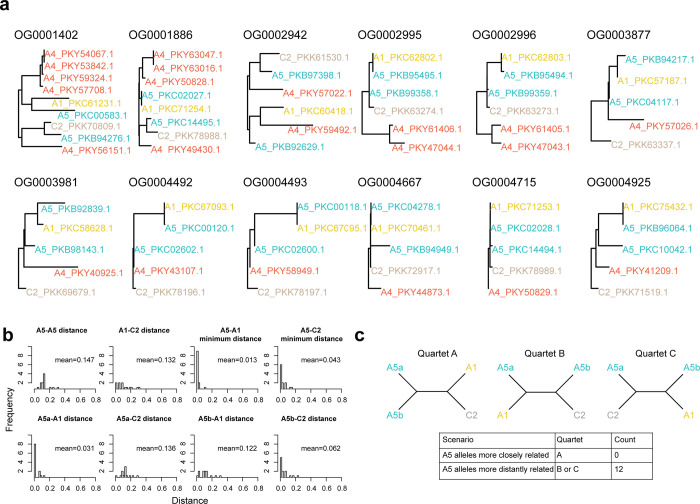
Nuclei from isolate A5 share a more recent evolutionary origin to isolates A1 and C2 than among them. **a,** Phylogenetic reconstruction of nucleotype-specific alleles in isolate A5 and its orthologs in isolates A1, C2 and A4. **c,** Average genetic distances between the different nucleotype-specific alleles from two alleles from isolates A5 and their homologs in isolates A1 and C2. We show histograms representing the genetic distance between the two nucleotype-specific alleles of isolate A5 and their homologues in isolates A1 and C2. For comparisons between A5 and C1 or A1 we used the minimum distance of the two alleles from A5. **d**, Scenarios of genetic similarity between the two A5 alleles and alleles from A1 and C2.

To confirm the previous findings, for each previously defined nucleotype-specific gene, we compared the phylogenetic relationship of the two nucleotype-specific alleles in isolate A5 isolate and in isolates A1, C2 and A4. We observed that for several nucleotype-specific alleles, the allele from isolate A1 clustered with one of the alleles of isolate A5, but it was not always the case for isolate C2 (Fig 4A).

We then analyzed the genetic distance of each of the 12 nucleotype-specific genes independently between the two alleles of isolate A5 and the homologous allele in isolates A1 and C2. The mean nucleotide distance between the two A5 alleles was 0.147. The mean distance between A1 and C2 was 0.132. In contrast, the mean of the minimum distance between an allele of isolate A5 and isolate A1 was 0.013 and between A5 and C2 was 0.043 (Fig 4B). We did not observe any case where the two A5 alleles clustered together, instead we observed that for all 12 nucleotype-specific gene the two A5 alleles were more similar to the allele from isolate A1 or C2 (Fig 4C). The mean distances calculated between alleles in this study are much higher than average distances calculated on the whole genome between different isolates [50], reflecting our selection criteria for nucleotype-specific regions. As each of the A5 alleles was closer to A1 or C2, instead of the two A5 alleles being most similar, this indicates that the alleles of A5 share a more recent evolutionary origin with these monokaryons than the two alleles within A5.

### Identification of reciprocal recombination between nucleotype-specific haplotypes in isolate A5 using single-nucleus data

**Fig 5:**
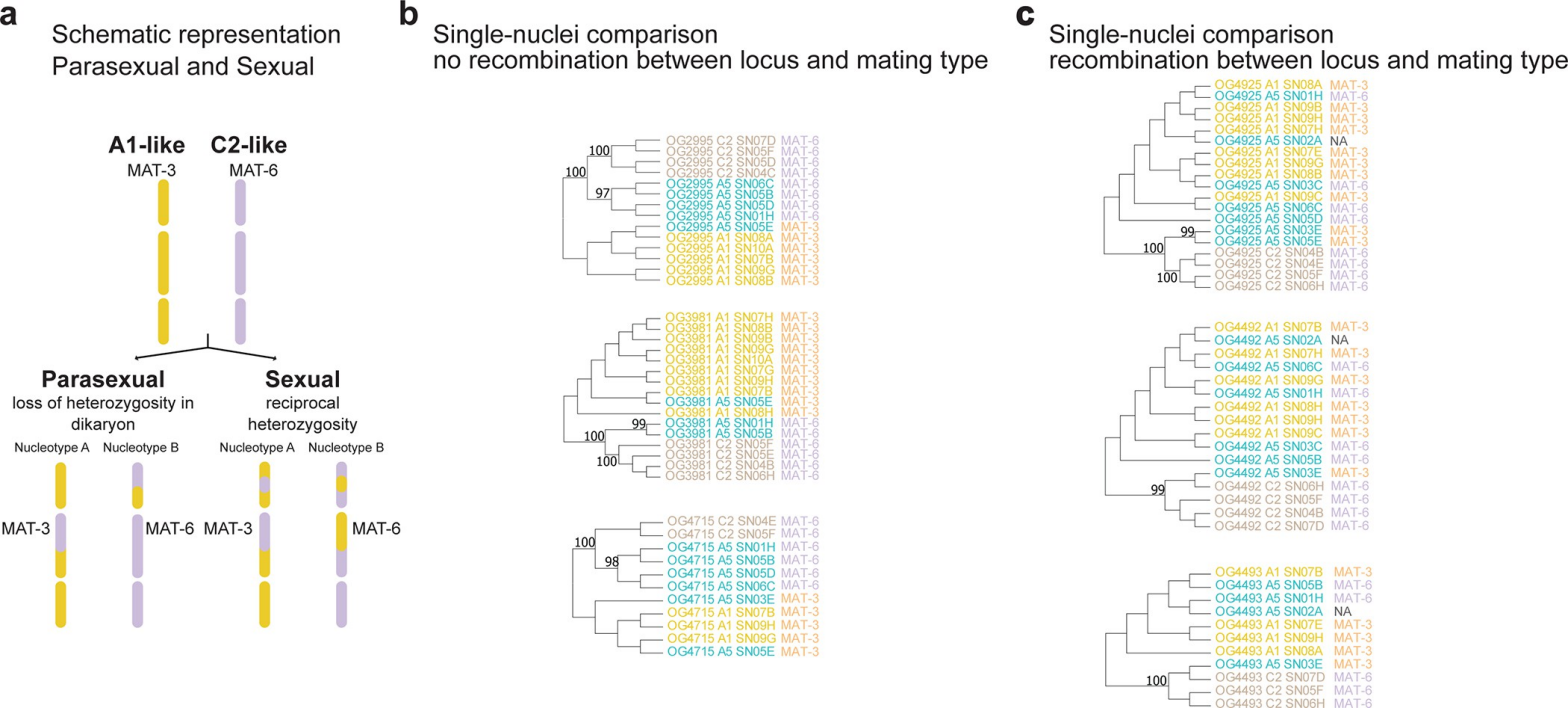
Recombination events in nucleotype-specific genes in A5 isolate. **a,** Schematic representation of possible outcomes after fusion of two different isolates. Please note the schema illustrates different contigs, separated by blank lines and no different chromosomes. **b,**Phylogenetic relationship of different nucleotype-specific alleles among different nuclei from A1, A5 and C2 isolates. Cases where no recombination was detected. Nuclei having the same MAT-type clustered together. **c**, Phylogenetic relationship of different nucleotype-specific alleles among different nuclei from A1, A5 and C2 isolates. Cases where recombination was detected. Nuclei having the same MAT-type did not clustered together. We performed 100 bootstraps for the branch support of all phylogenetic constructions.

Knowledge about nucleotype-specific alleles of dikaryon isolate A5 and their orthologs in isolates A1 and C2 allowed us to test whether parasexual or sexual genomic signatures could be identified in dikaryon isolate A5 (Fig 5A). We scanned the different nucleotype-specific-genes for the detection of recombination events within the two haplotypes of isolate A5. Comparison of gene sequences issued from the short-read whole genome assemblies from isolates A1, C2 and the two haplotypes of A5 showed that each nucleotype-specific allele from isolate A5 was highly similar to either the allele of isolate A1 or C2, but we did not identify any recombination events on these sequences (S4 Fig).

To further assess the potential for clonal relationships between the two alleles within A5 and isolates A1 and C2, we compared the nucleotype-specific alleles on the single-nucleus assemblies from isolate A5 and their orthologs on the single-nucleus assemblies of isolates A1 and C2 (S8 Table). The difference with the previous analysis is that with the single-nucleus data, we are able to identify the identity of the A5 alleles by identifying the identity of the putative MAT-type on each nucleus. We found that for several nucleotype-specific genes (i.e. OG2995, OG3981 and OG4715), the alleles from isolates A5 (MAT-3 type) and A1 (MAT-3 type) clustered together (S5 Fig, Fig 5B). However, we found that for other nucleotype-specific genes (OG4925, OG4492 and OG4493), the alleles from isolate A5 (MAT-3 type) clustered with the alleles from isolate C2 which has a MAT-6 type (Fig 5, S5 Fig, Fig 5C). The alignments on these nucleotype-specific genes show that A5 nuclei with MAT-3 have similar, but not identical alleles as C2 nuclei (MAT-6 type). In the same way, A5 nuclei with MAT-6 type harbor alleles similar to those of A1 nuclei (MAT-3 type). These results demonstrate that the A5 isolate harbors two nuclei genotypes, where one genotype is highly similar to the genotype displayed on nuclei of isolate A1 and the other A5 genotype is similar to the genotype of isolate C2. However, the A5 nuclei that is similar to the A1 nuclei, contain regions that are more similar to the equivalent region of isolate C2. This happens as well, for the A5 nuclei that is similar to isolate C2, where some regions are more similar to the equivalent region of isolate A1, demonstrating a reciprocal recombination pattern. The presence of reciprocal recombinant nuclei in isolate A5, involving isolates sharing the same MAT-type, strongly suggest that isolate A5 results from a sexual and not parasexual event between isolates similar to A1 and C2.

### Confirmation of reciprocal recombination events in isolate A5 using long-read genome assemblies

We compared single-copy orthologous genes among the A1, C2 assemblies and the phased assembly of isolate A5, which is divided in a primary assembly and an haplotig assembly.

The long-read assemblies were more complete and contain longer scaffolds than the short reads assemblies (S3B, S1C, S1E and S1F Fig). The A5 phased assembly consisted of a primary assembly of 392 scaffolds and an haplotig assembly on 292 of the primary scaffolds. The primary assembly was 115Mb long and the haplotig assembly was 19.2Mb [36].

**Fig 6:**
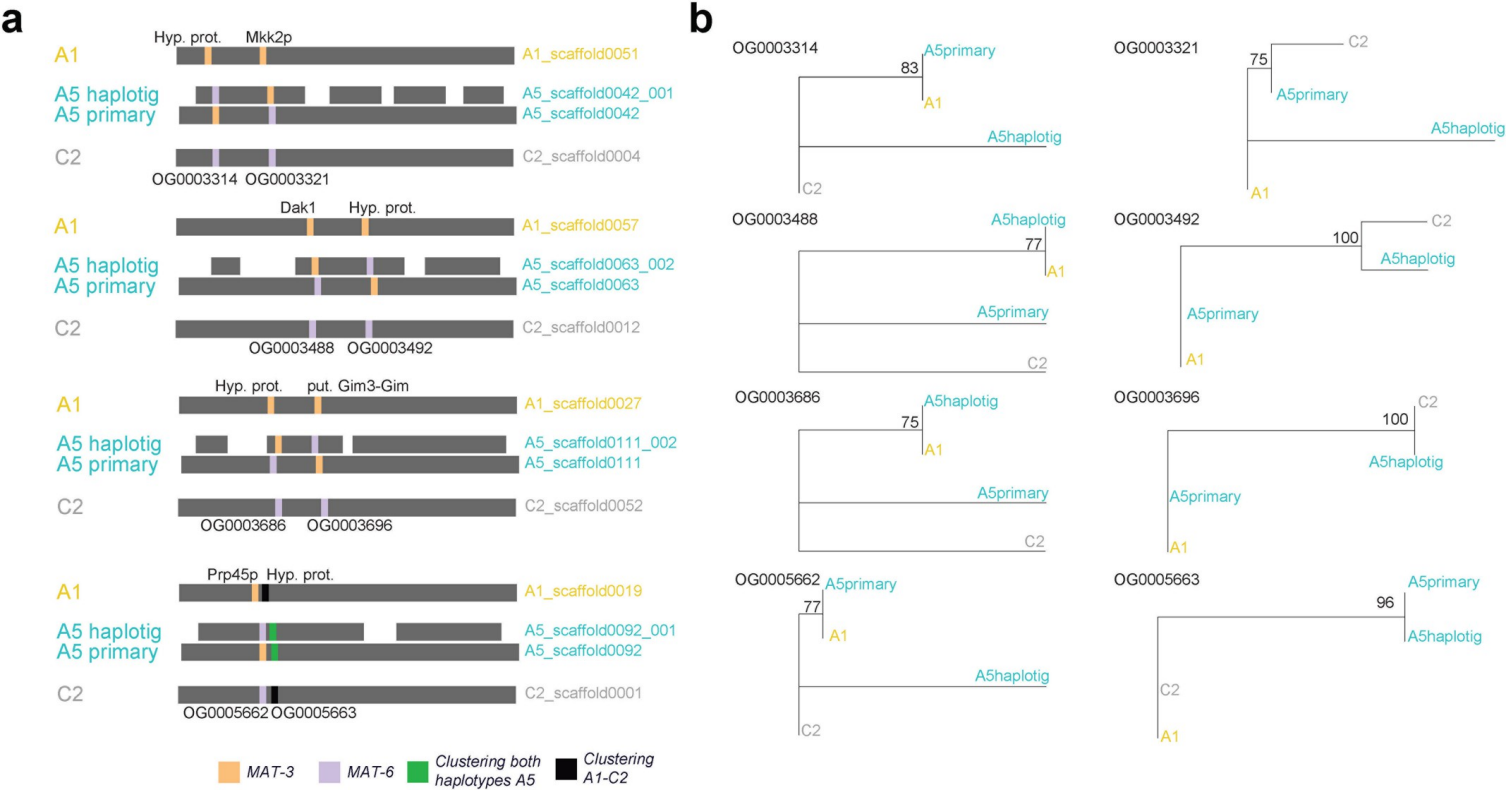
Recombination events identified in continuous haplotypes issued from the long-read genome assembly of dikaryon isolate A5. **a** Schematic representation of recombination events within continuous haplotypes. We highlight single-copy orthologous genes represented by different colors depending if they cluster with a gene of isolate A1 MAT-3 (yellow) or C2 MAT-6 (violet). Genetic recombination is demonstrated when within a continuous haplotype (grey background) a change in clustering pattern between an A5 haplotype and isolate A1 or C2 is observed. We can also observe that for few cases the two haplotypes of A5 clustered together (green). **b** Phylogenetic reconstructions of the different single-copy orthologous groups identified within the same haplotype. We can observe that the clustering pattern change within the same haplotype (i.e. A1-A5primary and C2-A5primary). We performed 100 bootstraps for the branch support of all phylogenetic constructions.

We identified 1250 single-copy orthologs among isolates A1, C2, the A5 primary assembly and the A5 haplotig assembly (S9 Table). We then focused only in the orthologs were one of the genes of A5 clustered with either A1 or C2. The majority of the gene sequences were very similar among all the isolates, however we identified 44 orthologous groups where one of the alleles of A5 isolate cluster with an allele of isolate A1 or C2 (23 genes A1-A5haplotig and 21 genes A1-A5primary). We observed that for several contiguous phased regions, a gene displayed the A5primary allele clustering with A1 allele and in another gene, contained in the same contiguous region, the A5 primary allele clustered with the C2 allele (Fig 6), confirming that reciprocal recombination signatures are found within continuous haplotypes (S9 Table).

## Discussion

In this study, we used published short and long read sequencing data to demonstrate that nucleotypes from the dikaryon isolate are more genetically related to monokaryon isolates displaying the same putative MAT-type than between each other. We also demonstrated that signatures of reciprocal recombination can be detected in genomes of dikaryon AMF isolates with the help of related monokaryon isolates that display the same putative MAT-type as the dikaryon. This study suggests that in the AMF life cycle there could be a transition between monokaryon and dikaryon isolates sharing the same putative MAT-type. It also suggests that a genetic exchange, involving reciprocal recombination, could be another mechanism generating genetic variability in AMF.

In the absence of experimental evidence that isolates with different putative MAT-types are involved in sexual reproduction, genomic signatures of recombination can help us to understand whether sexual or parasexual reproduction are involved in the AMF life cycle. On one hand, parasexual reproduction genomic signatures involves gene-conversion, which result is the loss of heterozygosity [51]. On the other hand, reciprocal recombination associated with a putative sex-determining region (MAT-type) is a genomic signature of sexual reproduction, although gene-conversion could also happen in sexual reproduction [52]. It could be plausible that parasexual recombination could lead to a reciprocal recombination pattern by fusion of two monokaryons, mitotic reciprocal recombination, followed by reciprocal chromosome loss, however this cascade of events seems less plausible.

In this manuscript, we used single-nucleus short read sequence data which produced highly fragmented and incomplete single-nucleus assemblies. We were able to work with this data because it served as a confirmatory dataset. We would not be able to identify recombination signatures relying only on the single-nucleus sequence dataset. The identification of genomic regions to test the hypothesis was made on the bulk short-read genome assemblies. However, with the bulk genome assemblies we were not able discriminate which of the two copies of the dikaryon is more similar to A1 or C2, because we did not have phasing information. It was at this step when the single-nucleus assemblies are useful. The single nucleus assemblies contained phasing information. Each nucleus had been sequenced independently and the information of the putative MAT-locus was associated to the nucleus ID. Consequently, there is an ID number (e.g. SN01) of a given nucleus and then its assembly is associated with that ID. Then the nucleus ID can be associated with the sequence of the putative MAT-locus. This makes it possible to associate other sequences at other genomic regions to the putative MAT-locus ID. We then were able to test our hypothesis by querying each nucleotype-specific gene detected with the bulk short-read data on the different types of nucleus (which are defined by their putative MAT locus).

We identified few regions that display recombination patterns within continuous haplotypes in the dikaryon isolate A5. The principal reason, is that we did not evaluate all the different genes present, but only the genes that display only 1 copy in a haploid phased contig. We only compared single-copy orthologs among the different assemblies, to avoid the confounding effect of paralogy on our results. In consequence, the examples of reciprocal recombination shown in this study, are only a small subset of the total plausible recombination spots present in their genomes. With the information of nucleotype-specific haplotypes identified with the drop in coverage analysis, we found that nucleotypes of isolate A5 were as little 1% diverged from isolate A1 and 4% diverged from isolate C2, suggesting that isolates sharing the same MAT-type as the monokaryon isolates A1 and C2 are closely related ancestors from which the dikaryon A5 arose. Interestingly, with the long-read data, we identified several loci where the A1 and C2 genes were more closely related among them than to the alleles of isolate A5. This suggests, that there could be other evolutionary forces shaping AMF genome evolution. AMF genomes could be influenced by horizontal gene transfer from the host plant or bacteria [53] Transposable elements (TE) have been reported to be responsible of genome duplications, inversions, insertions and deletions [18, 54, 55]. In nature, AMF co-exist with different conspecifics and they co-exists as well with different host-plants. We could speculate, that gene trajectories within AMF populations could be also driven by TE-mediated horizontal gene transfer events. However, an important sampling of AMF genomes is necessary to better understand the genome architecture features and life-history of the different gene families [56].

An intriguing question, is how only two coexisting nucleotypes and a well-orchestrated mechanism such as meiosis could be achieved without the presence of a single-cell stage in the AMF life cycle. A genetic bottleneck, where only 1 or 2 nuclei co-occur has never been detected in AMF. Despite the last feature, two studies that evaluated nuclear imbalance in dikaryon isolates show that nuclei ratio among single-spore lines are conserved despite multiple culturing for years [57] or are stable when exposed to different host plants [26]. These studies suggest that a nuclei regulation mechanism should exist and could be responsible of the stability of the nucleotypes in the dikaryons.

In this study, we compared haplotypes within the dikaryon A5 to monokaryon isolates (A1 and C2) that contain the same putative MAT-types as the dikaryon A5 to identify the recombination patterns. We were able to analyze these signatures on the A5 dikaryon, but not on the dikaryon A4, because there are no available genomic assemblies of monokaryon isolates displaying the same putative MAT-type as in Isolate A4. We hypothesized that MAT-type compatibility exists between the two different putative MAT-types identified in the dikaryon isolates. However, to date there is no direct experimental evidence that different putative MAT-types could be compatible. An indirect evidence of compatibility between different putative MAT-types was found, when two different isolates harboring different putative MAT-types elicited a putative fungal mating response [22, 23]. However, Mateus et al., did not test if the mating of two co-existing isolates took place [22]. Consequently, in order to experimentally identify mating and MAT-type compatibility, crossing experiments between strains harboring different putative MAT-types, including transcriptome and recombinant progeny analyses should be performed.

One of the main findings in this study is that in dikaryon short-read assemblies, if the two alleles of the same gene are highly divergent (i.e. like idiomorphs), the alleles are dispatched in two separate contigs. One allele can be found in a large contig containing the neighboring region of the allele and displaying a drop in coverage, whereas the second allele, without its neighboring region, is present in another short contig. We were able to demonstrate this by looking at the orthologous relationships between the two alleles of the gene present in the two different contigs. Our approach is reliable because we also detect the putative MAT-locus which is present in two separate contigs in the Illumina assemblies.

Our “drop in coverage” approach allowed us to identify divergent nucleotype-specific alleles in dikaryon isolates situated in different contigs of the short-read whole genome assemblies. This approach differs from previous approaches of global intra-isolate divergence assessment that measured the number of SNPs [58] or poly-allelic sites [9]. Although the comparison of both types of measurements gave similar information (intra-isolate divergence), their comparison should be carefully addressed as their methodology and the types of sequences compared are different. While SNPs are best identified in low divergence regions, where reads can be confidently mapped to the same contig, the sequences with large genetic differences, detected with the drop in coverage approach, are highly divergent to the point that they are assembled in different contigs in the same genome assembly. Consequently, genetic divergence between alleles detected in different genomic locations should be higher than when the two alleles are collapsed in the genome assembly. Contrary, to the drop in coverage analysis using short-read data, the analysis made on phased haplotypes from long-read sequencing, display genes that differ in several SNPs between the two dikaryon haplotypes.

Inter-nucleus recombination has been previously reported [24], although the robustness of the analysis has been questioned. The application of strict filtering parameters such as removal of heterozygous sites in haploid nuclei, duplicated regions of the genome, and low-coverage depths base calls results in an extreme loss of the signal of recombination [59]. Although some of these limitations, as coverage depth and filtering-out heterozygous sites were addressed [58], other limitations such as replicability (recombination events shown in several nuclei sharing the same putative MAT-type) and issues related to the whole-genome amplification step as the formation of chimeric sequences [60], allelic drop-out [61] and SNP miscalling [62] are inherent limitations of the analysis of any single nucleus amplification data. Furthermore, the previously reported evidence of large inter-nuclei recombination [24, 58] does not fit the observations about a monokaryon–dikaryons organization in AMF. In Chen *et al*., 2018a, the selected examples of genotypes presented, show 4 nuclei per isolate, which represent four different nucleus genotypes for SL1, A4 and three different nucleus genotypes on A5. The same pattern can be observed in Chen *et al*., 2020, the examples of genotypes show 7 out of 8 different nucleus genotypes in isolate A5, at least 7 different nucleus genotypes in isolate A4 and at least 5 different nucleus genotypes in SL1. The presence of repeated inter-nuclear recombination in dikaryons without prior crossing with other isolates, as observed in the single-nucleus genotypes shown in Chen et al. (2018a, 2020) would result in heterokaryons with more than two types of nucleus genotypes. But this does not appear to be the case for *R*. *irregularis* [16, 24, 59, 63].

Maintaining monokaryon and dikaryon isolates within the same natural population suggests that both forms are stable over time. Rather than a promiscuous mixing between isolates via anastomosis, a mechanism of recognition that involves a putative MAT-locus could regulate which isolates can form a dikaryon [64]. The fact that nucleotype-specific haplotypes from isolate A5 are more closely related to isolates A1 and C2, and that A5 nucleotypes display recombination, suggests that isolates sharing the same putative MAT-type as A1 and C2 could be the origin of a recombining dikaryon isolate. However, we cannot discard that these findings could apply to another step of the AMF life cycle. It could also be possible that a stable A5 isolate could segregate producing recombined monokaryons that share the same putative MAT-type as isolates A1 and C2, that can disperse and then fuse again to form stable dikaryons and complete a life cycle which involves recombination. It then becomes crucial to experimentally confirm if monokaryon isolates having different putative MAT-types could generate a dikaryon-like form and if a dikaryon isolate could segregate into recombining monokaryon isolates. It is difficult to establish *in-vitro* crosses mainly due to the difficulty of spore identification arising from the hyphae that fuse from the two parental isolates (an *in-vitro* culture produces thousands of spores). Hyphal fusion between different parental isolates has been reported [29, 65, 66]. We think that with technologies such as microfluidic channels developed for the study of filamentous fungi [67], it could be easier to identify hyphal fusions and the resulting spores from individual fusions. However, if recombination is a rare event between AMF genomes, which is a possibility, then it may be difficult to observe it in the laboratory.

Understanding the life cycle of AMF could have an enormous impact in the generation of AMF genetic variability. The generation of diverse AMF monokaryons or dikaryons could be used to generate variants that enhance plant growth and have an enormous potential in agriculture [11].

## Supporting information

S1 Fig Number of copies of each orthologous group within each isolate. Number of copies of different orthologous groups found within the genome of each isolate.
(PDF)

S2 Fig Synteny among candidate nucleotype specific regions in isolates A1, A4, A5 and C2. **a, b** We show examples of orthologous genes on different isolates that are situated in the same genomic location in isolate A4 and isolate A5 respectively. This is evidenced by the blast homology (Grey zones linking the different contigs) shown in surrounding regions of the focal gene (in red). **c, d** Examples of paralogous genes on different isolates where there genomic location of the gene is not the same. Evidenced by the lack of homology (grey zones linking the different contigs) of the surrounding regions of the focal gene (in red).
(PDF)

S3 Fig Total length and N50 statistics of the genome assemblies used in this study. **a, d** Single-nuclei assemblies, **b, e** short-reads assemblies, **c, f** long-reads genome assemblies.
(PDF)

S4 Fig Multiple sequence alignment of nucleotype-specific alleles of isolate A5 and isolates A1 and C2. Sequences are issued from the short-reads genome assemblies. The sequences shown are collapsed and do not represent the total length of the genes.
(PDF)

S5 Fig Multiple sequence alignment of nucleotype-specific alleles of single-nuclei of isolate A5 and isolates A1 and C2. This example represents the case when no recombination is identified. Please note that MAT-3 or MAT-6 sequences cluster together. The sequences are issued from the single-nuclei genome assemblies. The sequences shown are collapsed and do not represent the total length of the genes.
(PDF)

S6 Fig Multiple sequence alignment of nucleotype-specific alleles of single-nuclei of isolate A5 and isolates A1 and C2. This example represents the case when recombination is identified. Please note that MAT-3 or MAT-6 sequences do not cluster together. The sequences are issued from the single-nuclei genome assemblies. The sequences shown are collapsed and do not represent the total length of the genes.
(PDF)

S1 Table Public available data used in this study. (XLSX)

S2 Table Drop in coverage regions. (XLSX)

S3 Table Genes present in drop in coverage regions. (XLSX)

S4 Table Orthology inference among isolates A1,A4,A5 and C2. (XLSX)

S5 Table Putative nucleotype specific genes in isolate A4 and their orthologs in isolates A1,A5 and C2. (XLSX)

S6 Table Total length, number of contigs and N50 value of the different de novo single-nuclei genome assemblies. (XLSX)

S7 Table Confirmation of equivalent nucleotype specific genes between bulk-sequencing data and single-nuclei data on isolates A4 and A5. (XLSX)

S8 Table Confirmation of recombinant nucleotype specific genes in single-nuclei data from isolates A1, C2 and A5. (XLSX)

S9 Table Identification of single-copy orthologues in long-reads data and recombination examples. (XLSX)

S1 File Script used for the identification of drop in coverage regions. (TXT)

## References

[1] Harrison MJ. The arbuscular mycorrhizal symbiosis: An underground association. Trends Plant Sci. 1997;2: 54–60. doi: 10.1016/S1360-1385(97)82563-0

[2] van der Heijden M, Klironomos J, Ursic M, Moutoglis P, Streitwolf-Engel R, Boller T, et al. Mycorrhizal fungal diversity determines plant biodiversity, ecosystem variability and productivity. Nature. 1998;74: 69–72. Available: http://www.nature.com/nature/journal/v396/n6706/abs/396069a0.html

[3] Antunes PM, Koch AM, Morton JB, Rillig MC, Klironomos JN. Evidence for functional divergence in arbuscular mycorrhizal fungi from contrasting climatic origins. New Phytol. 2011;189: 507–14. doi: 10.1111/j.1469-8137.2010.03480.x 20880038

[4] Thygesen K, Larsen J, Bødker L. Arbuscular mycorrhizal fungi reduce development of pea root-rot caused by Aphanomyces euteiches using oospores as pathogen inoculum. Eur J Plant Pathol. 2004;110: 411–419. doi: 10.1023/B:EJPP.0000021070.61574.8b

[5] Augé RM. Water relations, drought and vesicular-arbuscular mycorrhizal symbiosis. Mycorrhiza. 2001;11: 3–42. doi: 10.1007/s005720100097

[6] Smith SE, Read DJ. Mycorrhizal Symbiosis. Third edit. London: Elsevier Ltd; 2008.

[7] Koch AM, Sanders IR, Kuhn G, Fontanillas P, Fumagalli L. High genetic variability and low local diversity in a population of arbuscular mycorrhizal fungi. PNAS. 2004;101: 2369–2374. doi: 10.1073/pnas.0306441101 14983016 PMC356957

[8] Croll D, Wille L, Gamper H a, Mathimaran N, Lammers PJ, Corradi N, et al. Genetic diversity and host plant preferences revealed by simple sequence repeat and mitochondrial markers in a population of the arbuscular mycorrhizal fungus Glomus intraradices. New Phytol. 2008;178: 672–87. doi: 10.1111/j.1469-8137.2008.02381.x 18298433

[9] Wyss T, Masclaux FG, Rosikiewicz P, Pagni M, Sanders IR. Population genomics reveals that within-fungus polymorphism is common and maintained in populations of the mycorrhizal fungus Rhizophagus irregularis. ISME J. 2016;10: 2514–2526. doi: 10.1038/ismej.2016.29 26953600 PMC5030683

[10] Angelard C, Colard A, Niculita-Hirzel H, Croll D, Sanders IR. Segregation in a mycorrhizal fungus alters rice growth and symbiosis-specific gene transcription. Curr Biol. 2010;20: 1216–21. doi: 10.1016/j.cub.2010.05.031 20541408

[11] Sanders IR. Designer mycorrhizas: Using natural genetic variation in AM fungi to increase plant growth. ISME J. 2010;4: 1081–1083. doi: 10.1038/ismej.2010.109 20613789

[12] Spatafora JW, Chang Y, Benny GL, Lazarus K, Smith ME, Berbee ML, et al. A phylum-level phylogenetic classification of zygomycete fungi based on genome-scale data. Mycologia. 2016;108: 1028–1046. doi: 10.3852/16-042 27738200 PMC6078412

[13] Remy W, Taylor TN, Hass H, Kerp H. Four hundred-million-year-old vesicular arbuscular mycorrhizae. Proc Natl Acad Sci U S A. 1994;91: 11841–11843. doi: 10.1073/pnas.91.25.11841 11607500 PMC45331

[14] Marleau J, Dalpé Y, St-Arnaud M, Hijri M. Spore development and nuclear inheritance in arbuscular mycorrhizal fungi. BMC Evol Biol. 2011;11: 51. doi: 10.1186/1471-2148-11-51 21349193 PMC3060866

[15] Lin K, Limpens E, Zhang Z, Ivanov S, Saunders DGO, Mu D, et al. Single nucleus genome sequencing reveals high similarity among nuclei of an endomycorrhizal fungus. PLoS Genet. 2014;10: e1004078. doi: 10.1371/journal.pgen.1004078 24415955 PMC3886924

[16] Ropars J, Toro KS, Noel J, Pelin A, Charron P, Farinelli L, et al. Evidence for the sexual origin of heterokaryosis in arbuscular mycorrhizal fungi. Nat Microbiol. 2016;1: 16033. doi: 10.1038/nmicrobiol.2016.33 27572831

[17] Kobayashi Y, Maeda T, Yamaguchi K, Kameoka H, Tanaka S, Ezawa T, et al. The genome of Rhizophagus clarus HR1 reveals a common genetic basis for auxotrophy among arbuscular mycorrhizal fungi. BMC Genomics. 2018;19: 1–11. doi: 10.1186/s12864-017-4368-0 29914365 PMC6007072

[18] Morin E, Miyauchi S, San Clemente H, Chen ECH, Pelin A, Providencia I, et al. Comparative genomics of Rhizophagus irregularis, R. cerebriforme, R. diaphanus and Gigaspora rosea highlights specific genetic features in Glomeromycotina. New Phytol. 2019;222: 1584–1598. doi: 10.1111/nph.15687 30636349

[19] Judson OP, Normark BB. Ancient asexual scandals. Trends Ecol Evol. 1996;11: 41–46. doi: 10.1016/0169-5347(96)81040-8 21237759

[20] Halary S, Malik S-B, Lildhar L, Slamovits CH, Hijri M, Corradi N. Conserved meiotic machinery in Glomus spp., a putatively ancient asexual fungal lineage. Genome Biol Evol. 2011;3: 950–8. doi: 10.1093/gbe/evr089 21876220 PMC3184777

[21] Croll D, Sanders IR. Recombination in Glomus intraradices, a supposed ancient asexual arbuscular mycorrhizal fungus. BMC Evol Biol. 2009;9: 13. doi: 10.1186/1471-2148-9-13 19146661 PMC2630297

[22] Mateus ID, Rojas EC, Savary R, Dupuis C, Masclaux FG, Aletti C, et al. Coexistence of genetically different Rhizophagus irregularis isolates induces genes involved in a putative fungal mating response. ISME J. 2020. doi: 10.1038/s41396-020-0694-3 32514118 PMC7490403

[23] Mateus ID, Lee S, Sanders IR, Sanders IR. Co-existence of AMF with different putative MAT-alleles induces genes homologous to those involved in mating in other fungi: a reply to Malar et al. ISME J. 2021. doi: 10.1038/s41396-021-00979-x 33941891 PMC8319373

[24] Chen EC, Mathieu S, Hoffrichter A, Sedzielewska-Toro K, Peart M, Pelin A, et al. Single nucleus sequencing reveals evidence of inter-nucleus recombination in arbuscular mycorrhizal fungi. Elife. 2018;7: 1–17. doi: 10.7554/eLife.39813 30516133 PMC6281316

[25] Masclaux FG, Wyss T, Mateus-Gonzalez ID, Aletti C, Sanders IR. Variation in allele frequencies at the bg112 locus reveals unequal inheritance of nuclei in a dikaryotic isolate of the fungus Rhizophagus irregularis. Mycorrhiza. 2018;28: 369–377. doi: 10.1007/s00572-018-0834-z 29675619

[26] Kokkoris V, Chagnon P-L, Yildirir G, Clarke K, Goh D, MacLean AM, et al. Host identity influences nuclear dynamics in arbuscular mycorrhizal fungi. Curr Biol. 2021;31: 1531–1538.e6. doi: 10.1016/j.cub.2021.01.035 33545043

[27] Giovannetti M, Azzolini D, Citernesi AS. Anastomosis Formation and Nuclear and Protoplasmic Exchange in Arbuscular Mycorrhizal Fungi. Appl Environ Microbiol. 1999;65: 5571–5575. doi: 10.1128/AEM.65.12.5571-5575.1999 10584019 PMC91759

[28] Bever JD, Morton J. Heritable variation and mechanism of inheritance of spore shape within a population of Scutellosporea pellucida, An arbuscular mycorrhi. Am J Bot. 1999;86: 1209–1216. 10487808

[29] Croll D, Giovannetti M, Koch AM, Sbrana C, Ehinger M, Lammers PJ, et al. Nonself vegetative fusion and genetic exchange in the arbuscular mycorrhizal fungus Glomus intraradices. New Phytol. 2009;181: 924–37. doi: 10.1111/j.1469-8137.2008.02726.x 19140939

[30] Pontecorvo G. The Parasexual Cycle in Fungi. Annu Rev Microbiol. 1956;10: 393–400. doi: 10.1146/annurev.mi.10.100156.002141 13363369

[31] Andersen SL, Sekelsky J. Meiotic versus mitotic recombination: Two different routes for double-strand break repair. BioEssays. 2010;32: 1058–1066. doi: 10.1002/bies.201000087 20967781 PMC3090628

[32] Yildirir G, Malar C M, Kokkoris V, Corradi N. Parasexual and Sexual Reproduction in Arbuscular Mycorrhizal Fungi: Room for Both. Trends Microbiol. 2020;xx: 1–3. doi: 10.1016/j.tim.2020.03.013 32360097

[33] Helber N, Requena N. Expression of the fluorescence markers DsRed and GFP fused to a nuclear localization signal in the arbuscular mycorrhizal fungus Glomus intraradices. New Phytol. 2008;177: 537–48. doi: 10.1111/j.1469-8137.2007.02257.x 17995919

[34] Yoon S, Xuan Z, Makarov V, Ye K, Sebat J. Sensitive and accurate detection of copy number variants using read depth of coverage. Genome Res. 2009;19: 1586–1592. doi: 10.1101/gr.092981.109 19657104 PMC2752127

[35] Li F, Upadhyaya NM, Sperschneider J, Matny O, Nguyen-Phuc H, Mago R, et al. Emergence of the Ug99 lineage of the wheat stem rust pathogen through somatic hybridisation. Nat Commun. 2019;10: 5068. doi: 10.1038/s41467-019-12927-7 31699975 PMC6838127

[36] Chaturvedi A, Cruz Corella J, Robbins C, Loha A, Menin L, Gasilova N, et al. The methylome of the model arbuscular mycorrhizal fungus, Rhizophagus irregularis, shares characteristics with early diverging fungi and Dikarya. Commun Biol. 2021;4: 901. doi: 10.1038/s42003-021-02414-5 34294866 PMC8298701

[37] Leinonen R, Sugawara H, Shumway M. The sequence read archive. Nucleic Acids Res. 2011;39: 2010–2012. doi: 10.1093/nar/gkq1019 21062823 PMC3013647

[38] Krueger F. Trim galore. A wrapper tool around Cutadapt FastQC to consistently apply Qual Adapt trimming to FastQ files. 2015;516: 517.

[39] Li H. Aligning sequence reads, clone sequences and assembly contigs with BWA-MEM. 2013;00: 1–3. Available: http://arxiv.org/abs/1303.3997

[40] Quinlan AR, Hall IM. BEDTools: A flexible suite of utilities for comparing genomic features. Bioinformatics. 2010;26: 841–842. doi: 10.1093/bioinformatics/btq033 20110278 PMC2832824

[41] Bankevich A, Nurk S, Antipov D, Gurevich AA, Dvorkin M, Kulikov AS, et al. SPAdes: A new genome assembly algorithm and its applications to single-cell sequencing. J Comput Biol. 2012;19: 455–477. doi: 10.1089/cmb.2012.0021 22506599 PMC3342519

[42] Mikheenko A, Prjibelski A, Saveliev V, Antipov D, Gurevich A. Versatile genome assembly evaluation with QUAST-LG. Bioinformatics. 2018;34: i142–i150. doi: 10.1093/bioinformatics/bty266 29949969 PMC6022658

[43] Camacho C, Coulouris G, Avagyan V, Ma N, Papadopoulos J, Bealer K, et al. BLAST+: Architecture and applications. BMC Bioinformatics. 2009;10: 1–9. doi: 10.1186/1471-2105-10-421 20003500 PMC2803857

[44] Emms DM, Kelly S. OrthoFinder: Phylogenetic orthology inference for comparative genomics. Genome Biol. 2019;20: 1–14. doi: 10.1186/s13059-019-1832-y 31727128 PMC6857279

[45] Sullivan MJ, Petty NK, Beatson SA. Easyfig: A genome comparison visualizer. Bioinformatics. 2011;27: 1009–1010. doi: 10.1093/bioinformatics/btr039 21278367 PMC3065679

[46] Katoh K, Rozewicki J, Yamada KD. MAFFT online service: multiple sequence alignment, interactive sequence choice and visualization. Brief Bioinform. 2017; 1–7. doi: 10.1093/bib/bbx108 28968734 PMC6781576

[47] Paradis E, Claude J, Strimmer K. APE: Analyses of phylogenetics and evolution in R language. Bioinformatics. 2004;20: 289–290. doi: 10.1093/bioinformatics/btg412 14734327

[48] Kumar S, Stecher G, Li M, Knyaz C, Tamura K. MEGA X: Molecular evolutionary genetics analysis across computing platforms. Mol Biol Evol. 2018;35: 1547–1549. doi: 10.1093/molbev/msy096 29722887 PMC5967553

[49] Savary R, Masclaux FG, Wyss T, Droh G, Cruz Corella J, Machado AP, et al. A population genomics approach shows widespread geographical distribution of cryptic genomic forms of the symbiotic fungus Rhizophagus irregularis. ISME J. 2018;12: 17–30. doi: 10.1038/ismej.2017.153 29027999 PMC5739010

[50] Chen ECH, Morin E, Beaudet D, Noel J, Yildirir G, Ndikumana S, et al. High intraspecific genome diversity in the model arbuscular mycorrhizal symbiont Rhizophagus irregularis. New Phytol. 2018;220: 1161–1171. doi: 10.1111/nph.14989 29355972

[51] Forche A, Alby K, Schaefer D, Johnson AD, Berman J, Bennett RJ. The Parasexual Cycle in Candida albicans Provides an Alternative Pathway to Meiosis for the Formation of Recombinant Strains. Heitman J, editor. PLoS Biol. 2008;6: e110. doi: 10.1371/journal.pbio.0060110 18462019 PMC2365976

[52] Sun S, Hsueh YP, Heitman J. Gene conversion occurs within the mating-type locus of Cryptococcus neoformans during sexual reproduction. PLoS Genet. 2012;8. doi: 10.1371/journal.pgen.1002810 22792079 PMC3390403

[53] Li M, Zhao J, Tang N, Sun H, Huang J. Horizontal Gene Transfer From Bacteria and Plants to the Arbuscular Mycorrhizal Fungus Rhizophagus irregularis. 2018;9: 1–13. doi: 10.3389/fpls.2018.00701 PMC5982333 29887874

[54] Daboussi M-J, Capy P. Transposable Elements in Filamentous Fungi. Annu Rev Microbiol. 2003;57: 275–299. doi: 10.1146/annurev.micro.57.030502.091029 14527280

[55] Venice F, Ghignone S, Salvioli A, Amselem J, Novero M, Xianan X, et al. At the nexus of three kingdoms: the genome of the mycorrhizal fungus Gigaspora margarita provides insights into plant, endobacterial and fungal interactions. 2020;22: 122–141. doi: 10.1111/1462-2920.14827 31621176

[56] Badet T, Croll D. The rise and fall of genes: origins and functions of plant pathogen pangenomes. Curr Opin Plant Biol. 2020;56: 65–73. doi: 10.1016/j.pbi.2020.04.009 32480355

[57] Robbins C, Cruz Corella J, Aletti C, Seiler R, Mateus ID, Lee S, et al. Generation of disproportionate nuclear genotype proportions in Rhizophagus irregularis progeny causes allelic imbalance in gene transcription. New Phytol. 2021. doi: 10.1111/nph.17530 PMC8457141 34085297

[58] Chen ECH, Mathieu S, Hoffrichter A, Ropars J, Dreissig S, Fuchs J, et al. More Filtering on SNP Calling Does Not Remove Evidence of Inter-Nucleus Recombination in Dikaryotic Arbuscular Mycorrhizal Fungi. Front Plant Sci. 2020;11: 1–9. doi: 10.3389/fpls.2020.00912 32733503 PMC7358544

[59] Auxier B, Bazzicalupo A. Comment on “Single nucleus sequencing reveals evidence of inter-nucleus recombination in arbuscular mycorrhizal fungi.” Elife. 2019;8: 1–9. doi: 10.7554/eLife.47301 31650958 PMC6814362

[60] Yilmaz S, Singh AK. Single cell genome sequencing. Curr Opin Biotechnol. 2012;23: 437–43. doi: 10.1016/j.copbio.2011.11.018 22154471 PMC3318999

[61] Lauri A, Lazzari G, Galli C, Lagutina I, Genzini E, Braga F, et al. Assessment of MDA efficiency for genotyping using cloned embryo biopsies. Genomics. 2013;101: 24–9. doi: 10.1016/j.ygeno.2012.09.002 22982297

[62] Ning L, Liu G, Li G, Hou Y, Tong Y, He J. Current challenges in the bioinformatics of single cell genomics. Front Oncol. 2014;4 JAN: 1–7. doi: 10.3389/fonc.2014.00007 24478987 PMC3902584

[63] Masclaux FG, Wyss T, Pagni M, Rosikiewicz P, Sanders IR. Investigating unexplained genetic variation and its expression in the arbuscular mycorrhizal fungus Rhizophagus irregularis: A comparison of whole genome and RAD sequencing data. PLoS One. 2019;14: 1–20. doi: 10.1371/journal.pone.0226497 31881076 PMC6934306

[64] Corradi N, Brachmann A. Fungal Mating in the Most Widespread Plant Symbionts? Trends Plant Sci. 2017;22: 175–183. doi: 10.1016/j.tplants.2016.10.010 27876487

[65] Avio L, Pellegrino E, Bonari E, Giovannetti M. Functional diversity of arbuscular mycorrhizal fungal isolates in relation to extraradical mycelial networks. 2002; 347–357. 10.1111/j.1469-8137.2006.01839.x 16995921

[66] Pepe A, Giovannetti M, Sbrana C. Different levels of hyphal self-incompatibility modulate interconnectedness of mycorrhizal networks in three arbuscular mycorrhizal fungi within the Glomeraceae. Mycorrhiza. 2016;26: 325–332. doi: 10.1007/s00572-015-0671-2 26630971

[67] Held M, Lee AP, Edwards C, Nicolau D V. Microfluidics structures for probing the dynamic behaviour of filamentous fungi. Microelectron Eng. 2010;87: 786–789. doi: 10.1016/j.mee.2009.11.096

