# Supplementary figures and images for: Reciprocal recombination genomic signatures in the symbiotic arbuscular mycorrhizal fungi Rhizophagus irregularis
Source: PLoS One. 2022 Jul 1;17(7):e0270481. doi: 10.1371/journal.pone.0270481 (PMC9249182; doi:10.1371/journal.pone.0270481)

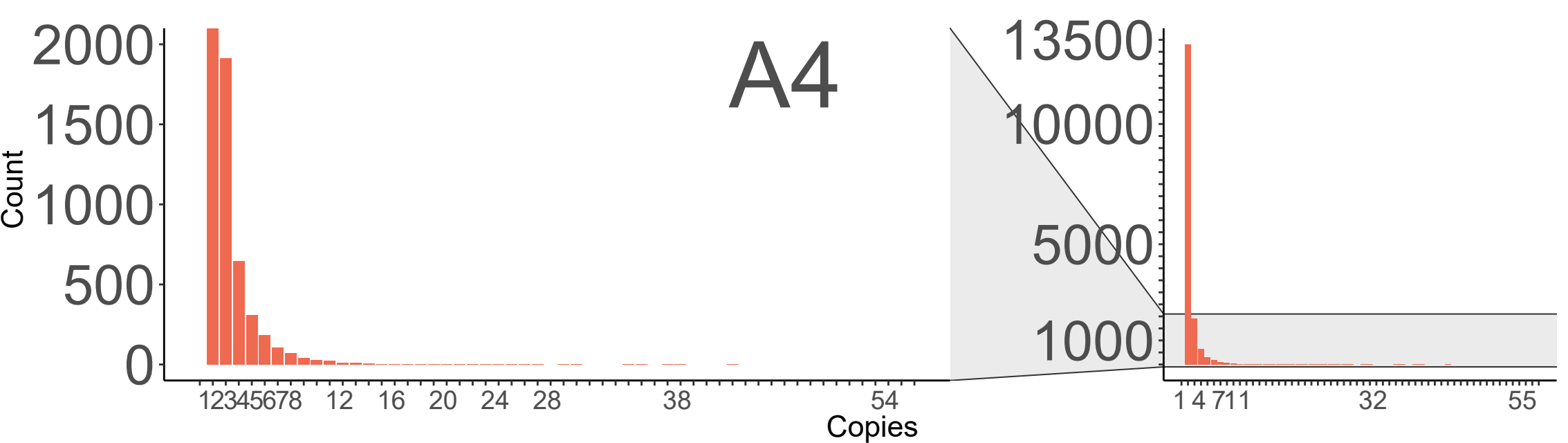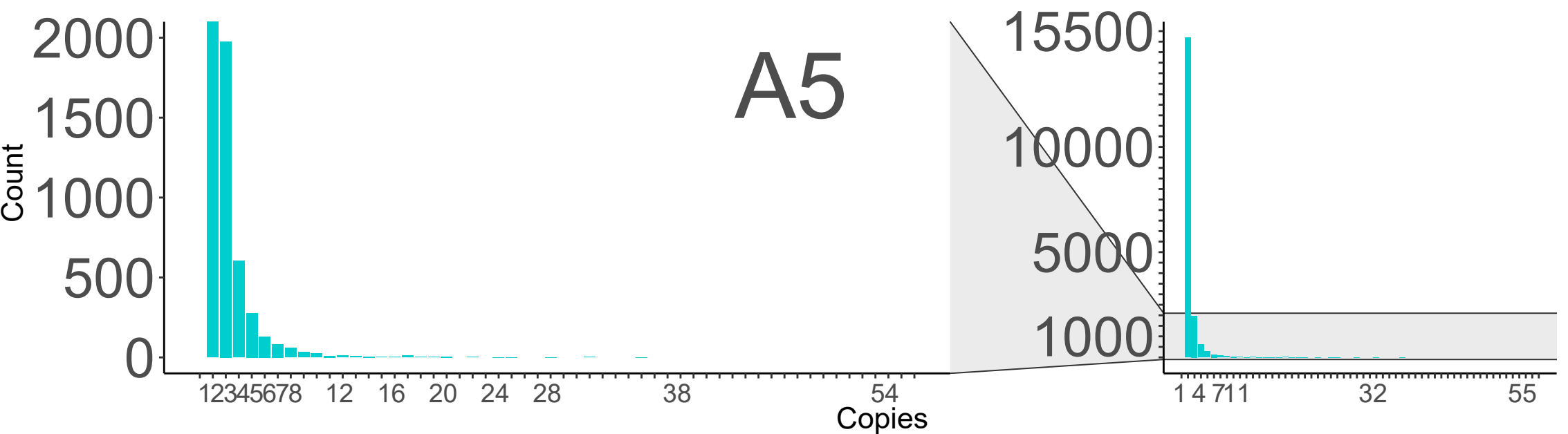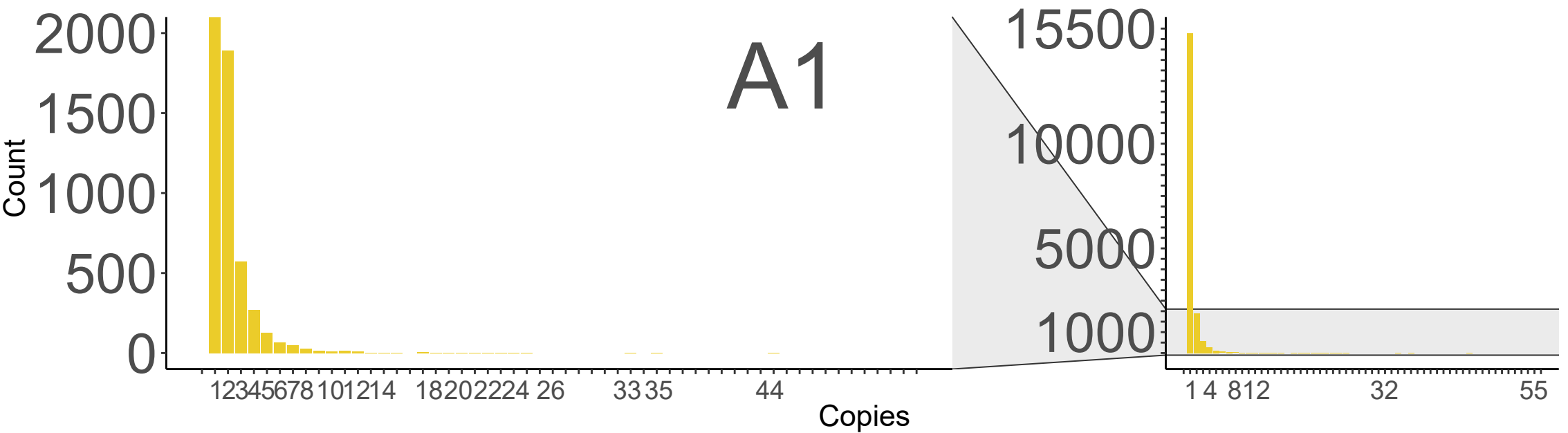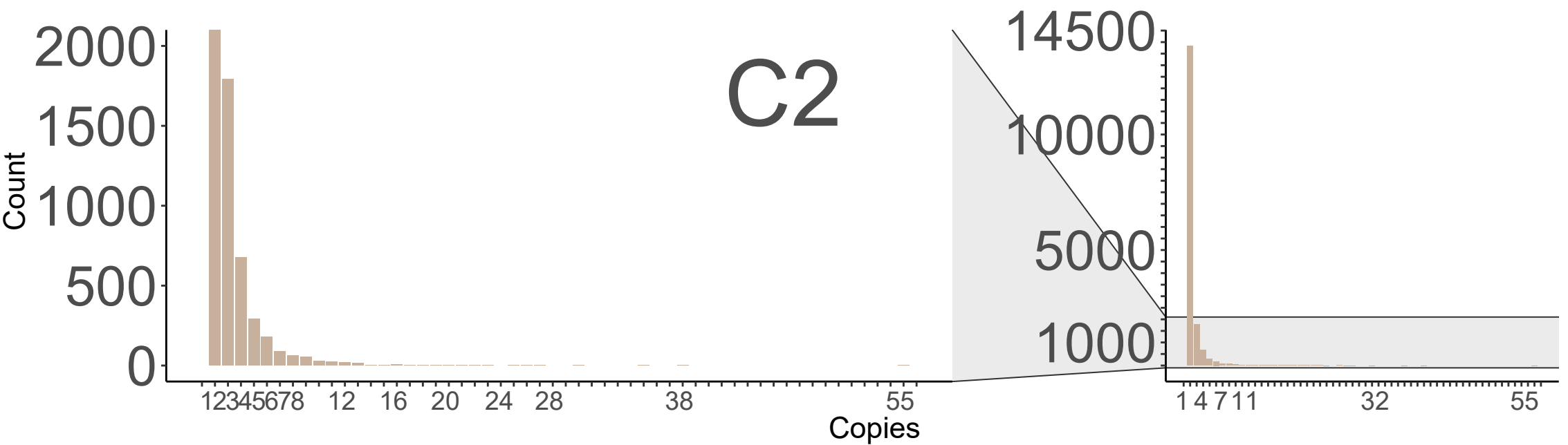

Supplement: S1 Fig — Number of copies of different orthologous groups found within the genome of each isolate. (PDF) [file pone.0270481.s001.pdf]

Whole-genome assemblies comparison

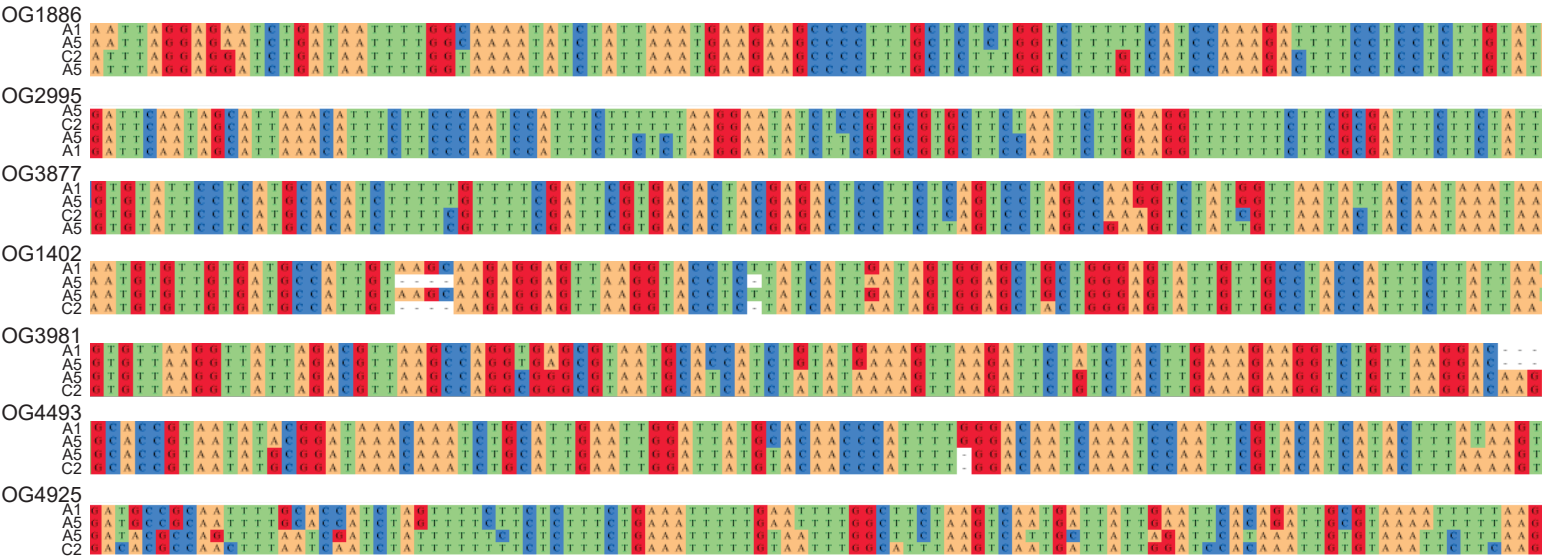

Supplement: S4 Fig — Sequences are issued from the short-reads genome assemblies. The sequences shown are collapsed and do not represent the total length of the genes. (PDF) [file pone.0270481.s004.pdf]
