# Supplementary material for: Reciprocal recombination genomic signatures in the symbiotic arbuscular mycorrhizal fungi Rhizophagus irregularis
Source: PLoS One. 2022 Jul 1;17(7):e0270481. doi: 10.1371/journal.pone.0270481 (PMC9249182; doi:10.1371/journal.pone.0270481)
Supplement: S2 Fig — a, b We show examples of orthologous genes on different isolates that are situated in the same genomic location in isolate A4 and isolate A5 respectively. This is evidenced by the blast homology (Grey zones linking the different contigs) shown in surrounding regions of the focal gene (in red). c, d Examples of paralogous genes on different isolates where there genomic location of the gene is not the same. Evidenced by the lack of homology (grey zones linking the different contigs) of the surrounding regions of the focal gene (in red). (PDF) [file pone.0270481.s002.pdf]

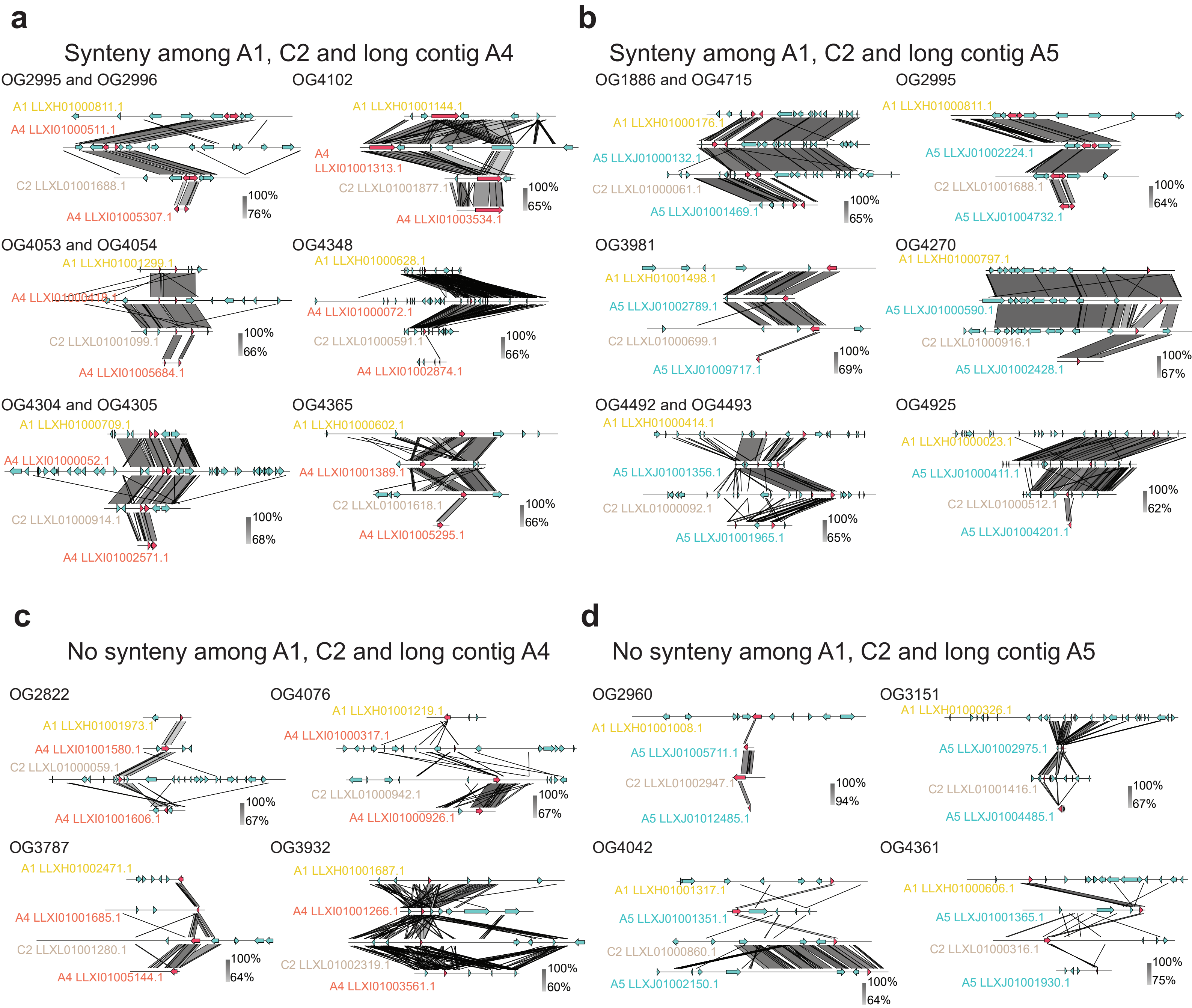
a
Synteny among A1, C2 and long contig A4
OG2995 and OG2996
A1 LLXH01000811.1
A4 LLXI01000511.1
C2 LLXL01001688.1
A4 LLXI01005307.1
OG4102
A1 LLXH01001144.1
A4 LLXI01001313.1
C2 LLXL01001877.1
A4 LLXI01003534.1
OG4053 and OG4054
A1 LLXH01001299.1
C2 LLXL01001099.1
A4 LLXI01005684.1
OG4348
A1 LLXH01000628.1
A4 LLXI01000072.1
C2 LLXL01000591.1
A4 LLXI01002874.1
OG4304 and OG4305
A1 LLXH01000709.1
A4 LLXI01000052.1
C2 LLXL01000914.1
A4 LLXI01002571.1
OG4365
A1 LLXH01000602.1
A4 LLXI01001389.1
C2 LLXL01001618.1
A4 LLXI01005295.1
b
Synteny among A1, C2 and long contig A5
OG1886 and OG4715
A1 LLXH01000176.1
A5 LLXJ01000132.1
C2 LLXL01000061.1
A5 LLXJ01001469.1
OG2995
A1 LLXH01000811.1
A5 LLXJ01002224.1
C2 LLXL01001688.1
A5 LLXJ01004732.1
OG3981
A1 LLXH01001498.1
A5 LLXJ01002789.1
C2 LLXL01000699.1
A5 LLXJ01009717.1
OG4270
A1 LLXH01000797.1
A5 LLXJ01000590.1
C2 LLXL01000916.1
A5 LLXJ01002428.1
OG4492 and OG4493
A1 LLXH01000414.1
A5 LLXJ01001356.1
C2 LLXL01000092.1
A5 LLXJ01001965.1
OG4925
A1 LLXH01000023.1
A5 LLXJ01000411.1
C2 LLXL01000512.1
A5 LLXJ01004201.1
c
No synteny among A1, C2 and long contig A4
OG2822
A1 LLXH01001973.1
A4 LLXI01001580.1
C2 LLXL01000059.1
A4 LLXI01001606.1
OG4076
A1 LLXH01001219.1
A4 LLXI01000317.1
C2 LLXL01000942.1
A4 LLXI01000926.1
OG3787
A1 LLXH01002471.1
A4 LLXI01001685.1
C2 LLXL01001280.1
A4 LLXI01005144.1
OG3932
A1 LLXH01001687.1
A4 LLXI01001266.1
C2 LLXL01002319.1
A4 LLXI01003561.1
d
No synteny among A1, C2 and long contig A5
OG2960
A1 LLXH01001008.1
A5 LLXJ01005711.1
C2 LLXL01002947.1
A5 LLXJ01012485.1
OG3151
A1 LLXH01000326.1
A5 LLXJ01002975.1
C2 LLXL01001416.1
A5 LLXJ01004485.1
OG4042
A1 LLXH01001317.1
A5 LLXJ01001351.1
C2 LLXL01000860.1
A5 LLXJ01002150.1
OG4361
A1 LLXH01000606.1
A5 LLXJ01001365.1
C2 LLXL01000316.1
A5 LLXJ01001930.1
100%
76%
65%
66%
68%
64%
69%
67%
62%
94%
60%
75%
