# Supplementary material for: Reciprocal recombination genomic signatures in the symbiotic arbuscular mycorrhizal fungi Rhizophagus irregularis
Source: PLoS One. 2022 Jul 1;17(7):e0270481. doi: 10.1371/journal.pone.0270481 (PMC9249182; doi:10.1371/journal.pone.0270481)
Supplement: S3 Fig — a, d Single-nuclei assemblies, b, e short-reads assemblies, c, f long-reads genome assemblies. (PDF) [file pone.0270481.s003.pdf]

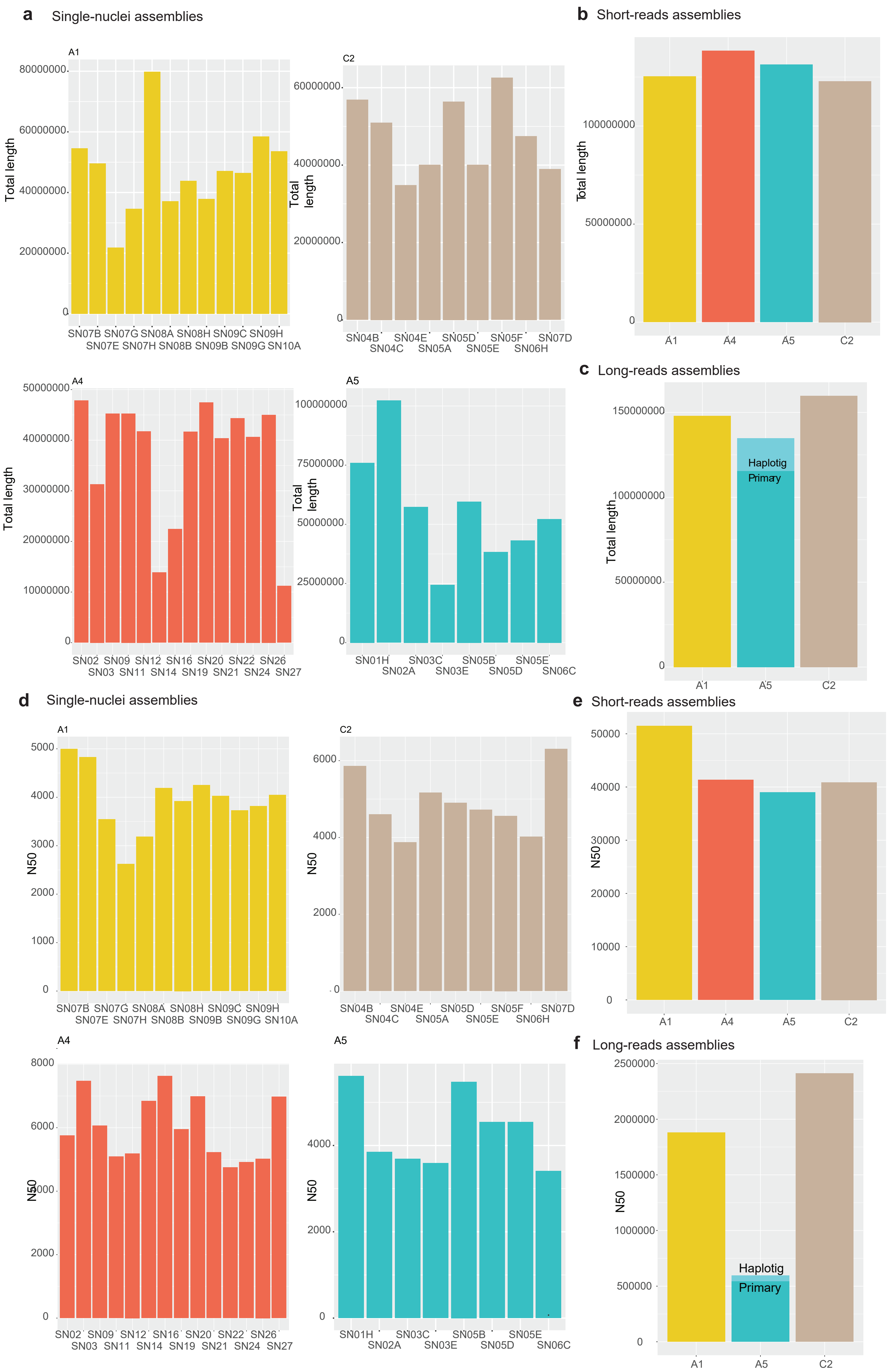
a
Single-nuclei assemblies
A1
Total length
80000000
60000000
40000000
20000000
0
SN07B SN07G SN08A SN08H SN09C SN09H
SN07E SN07H SN08B SN09B SN09G SN10A
C2
Total length
60000000
40000000
20000000
0
SN04B SN04E SN05D SN05F SN07D
SN04C SN05A SN05E SN06H
b
Short-reads assemblies
Total length
100000000
50000000
0
A1 A4 A5 C2
A4
Total length
50000000
40000000
30000000
20000000
10000000
0
SN02 SN09 SN12 SN16 SN20 SN22 SN26
SN03 SN11 SN14 SN19 SN21 SN24 SN27
A5
Total length
100000000
75000000
50000000
25000000
0
SN01H SN03C SN05B SN05E
SN02A SN03E SN05D SN06C
c
Long-reads assemblies
Total length
150000000
100000000
50000000
0
Haplotig
Primary
A1 A5 C2
d
Single-nuclei assemblies
A1
N50
5000
4000
3000
2000
1000
0
SN07B SN07G SN08A SN08H SN09C SN09H
SN07E SN07H SN08B SN09B SN09G SN10A
C2
N50
6000
4000
2000
0
SN04B SN04E SN05D SN05F SN07D
SN04C SN05A SN05E SN06H
e
Short-reads assemblies
N50
50000
40000
30000
20000
10000
0
A1 A4 A5 C2
A4
N50
8000
6000
4000
2000
0
SN02 SN09 SN12 SN16 SN20 SN22 SN26
SN03 SN11 SN14 SN19 SN21 SN24 SN27
A5
N50
4000
2000
0
SN01H SN03C SN05B SN05E
SN02A SN03E SN05D SN06C
f
Long-reads assemblies
N50
2500000
2000000
1500000
1000000
500000
0
Haplotig
Primary
A1 A5 C2
