# Supplementary material for: Reciprocal recombination genomic signatures in the symbiotic arbuscular mycorrhizal fungi Rhizophagus irregularis
Source: PLoS One. 2022 Jul 1;17(7):e0270481. doi: 10.1371/journal.pone.0270481 (PMC9249182; doi:10.1371/journal.pone.0270481)

Single-nuclei comparison
recombination between locus and mating type

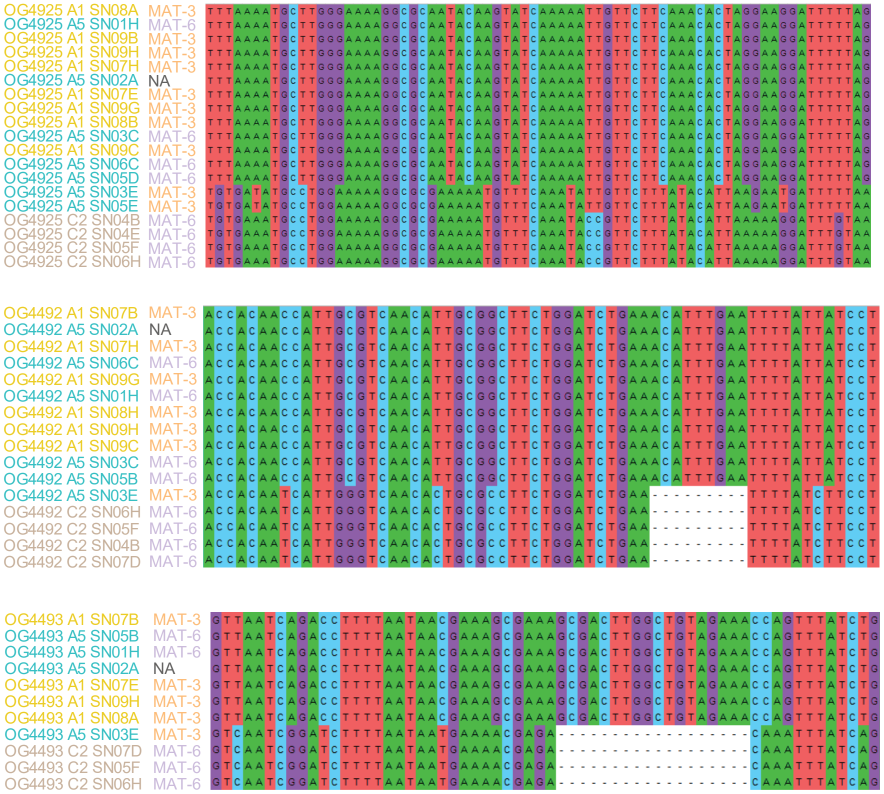

Supplement: S6 Fig — This example represents the case when recombination is identified. Please note that MAT-3 or MAT-6 sequences do not cluster together. The sequences are issued from the single-nuclei genome assemblies. The sequences shown are collapsed and do not represent the total length of the genes. (PDF) [file pone.0270481.s006.pdf]
